# Copper Promotes Tumorigenesis by Activating the PDK1‐AKT Oncogenic Pathway in a Copper Transporter 1 Dependent Manner

**DOI:** 10.1002/advs.202004303

**Published:** 2021-07-18

**Authors:** Jianping Guo, Ji Cheng, Nana Zheng, Xiaomei Zhang, Xiaoming Dai, Linli Zhang, Changjiang Hu, Xueji Wu, Qiwei Jiang, Depei Wu, Hitoshi Okada, Pier Paolo Pandolfi, Wenyi Wei

**Affiliations:** ^1^ Department of Pathology Beth Israel Deaconess Medical Center Harvard Medical School Boston MA 02215 USA; ^2^ Institute of Precision Medicine the First Affiliated Hospital Sun Yat‐sen University Guangzhou Guangdong 510275 China; ^3^ Department of Gastrointestinal Surgery Union Hospital Tongji Medical College Huazhong University of Science and Technology Wuhan Hubei 430022 China; ^4^ National Clinical Research Center for Hematologic Diseases Jiangsu Institute of Hematology The First Affiliated Hospital of Soochow University Suzhou Jiangsu 215000 China; ^5^ Department of Oncology Tongji Hospital, Tongji Medical College Huazhong University of Science and Technology Wuhan Hubei 430030 China; ^6^ Department of Biochemistry Kindai University Faculty of Medicine 377‐2 Ohno‐Higashi Osaka‐Sayama Osaka 589‐8511 Japan; ^7^ Division of Genetics Department of Medicine Beth Israel Deaconess Medical Center Harvard Medical School Boston MA 02215 USA

**Keywords:** AKT, breast cancer, copper, CTR1, Nedd4l, PDK1

## Abstract

Copper plays pivotal roles in metabolic homoeostasis, but its potential role in human tumorigenesis is not well defined. Here, it is revealed that copper activates the phosphoinositide 3‐kinase (PI3K)‐protein kinase B (PKB, also termed AKT) oncogenic signaling pathway to facilitate tumorigenesis. Mechanistically, copper binds 3‐phosphoinositide dependent protein kinase 1 (PDK1), in turn promotes PDK1 binding and subsequently activates its downstream substrate AKT to facilitate tumorigenesis. Blocking the copper transporter 1 (CTR1)‐copper axis by either depleting *CTR1* or through the use of copper chelators diminishes the AKT signaling and reduces tumorigenesis. In support of an oncogenic role for CTR1, the authors find that CTR1 is abnormally elevated in breast cancer, and is subjected by NEDD4 like E3 ubiquitin protein ligase (Nedd4l)‐mediated negative regulation through ubiquitination and subsequent degradation. Accordingly, Nedd4l displays a tumor suppressive function by suppressing the CTR1‐AKT signaling. Thus, the findings identify a novel regulatory crosstalk between the Nedd4l‐CTR1‐copper axis and the PDK1‐AKT oncogenic signaling, and highlight the therapeutic relevance of targeting the CTR1‐copper node for the treatment of hyperactive AKT‐driven cancers.

## Graphical abstract

Besides playing pivotal roles in metabolic homoeostasis, copper also binds PDK1 to activate its downstream AKT kinase, which in turn facilitates tumorigenesis. Meanwhile, the copper transporter 1 (CTR1) is negatively regulated by Nedd4l‐mediated ubiquitination and subsequent degradation, indicating a novel regulatory crosstalk between the Nedd4l‐CTR1‐copper axis and the PDK1‐AKT oncogenic signaling to govern tumorigenesis.

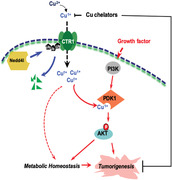

## 1 Introduction

Metal ions have been well‐established to play critical roles in the human body development, enzymatic activation, and metabolic homoeostasis.^[^ ^1^ ^]^ As such, abnormal homeostasis of metal ions pathologically contributes to a plethora of human disorders, including neurodegenerative diseases and cancers.^[^ ^1^, ^2^, ^3^ ^]^ For instance, calcium deficiency is associated with elevated tumorigenesis, whereas calcium and Vitamin D supplementation could reduce cancer incidences.^[^ ^4^, ^5^ ^]^ Moreover, elevated iron has been found to facilitate tumorigenesis in part by generating reactive oxidative species (ROS),^[^ ^6^ ^]^ while copper facilitates tumorigenesis by promoting tumor angiogenesis,^[^ ^7^ ^]^ regulating autophagy via binding Unc‐51 like autophagy activating kinases (ULK1/2),^[^ ^8^ ^]^ or modulating cell growth via binding oncoproteins such as extracellular signal‐regulated kinase (MEK) and mediator of Erb‐B2 receptor tyrosine kinase 2‐driven cell motility (Memo), respectively.^[^ ^9^, ^10^ ^]^

It is well‐established that the hyperactivation of the PI3K‐PDK1‐AKT axis is a central module of cell proliferation, survival, drug‐resistance, and metabolic homeostasis in various human diseases, including diabetes and cancers.^[^ ^11^, ^12^ ^]^ Physiologically, stimulations derived from growth factors, cytokines, or inflammatory factors all prefer to activate AKT, in turn to phosphorylate its distinct downstream substrates to perform complicated biological processes.^[^ ^12^, ^13^ ^]^ More importantly, genetical alterations of the PI3K‐AKT signaling pathway including gain‐of‐function mutations of phosphatidylinositol‐4,5‐bisphosphate 3‐kinase catalytic subunit alpha (*PIK3CA*), *RAS*, and *AKT*, or deletion/loss‐of‐function mutations of phosphatase and tensin homolog (*PTEN*), neurofibromatosis type 1 (*NF1*), and Von Hippel‐Lindau syndrome (*VHL*) all confer to AKT kinase activity and oncogenic functions in tumorigenesis. Recently, tumor microenvironmental changes, such as deprivation of oxygen (hypoxia) or reactive oxygen species (ROS) stress have also been highlighted to activate AKT by distinct mechanisms,^[^ ^14^, ^15^ ^]^ however, the physiological roles of metal ions influencing AKT kinase activity and oncogenic functions are not fully defined.

In this report, via screening a panel of mental ions, we identify that copper could strongly activate AKT kinase in a copper transporter (CTR1)‐dependent manner. Mechanistically, copper could bind PDK1, to promote its interaction with AKT, and activate AKT oncogenic signaling in a PI3K dependent manner. Notably, we demonstrate that the major oncogenic function of CTR1 is via activating AKT kinase, and is undergoing Nedd4l‐mediated ubiquitination and subsequent degradation. All these findings together linearize the delicate regulation of AKT kinase by Nedd4l‐CTR1 signaling pathway in a copper‐PDK1 binding dependent manner, and highlight the potential strategy to combat hyperactive AKT‐driven cancers by targeting CTR1‐copper pathway.

## 2 Results

### 2.1 The CTR1‐Copper Axis Plays Important Roles in Activating AKT Kinase

**Figure 1:**
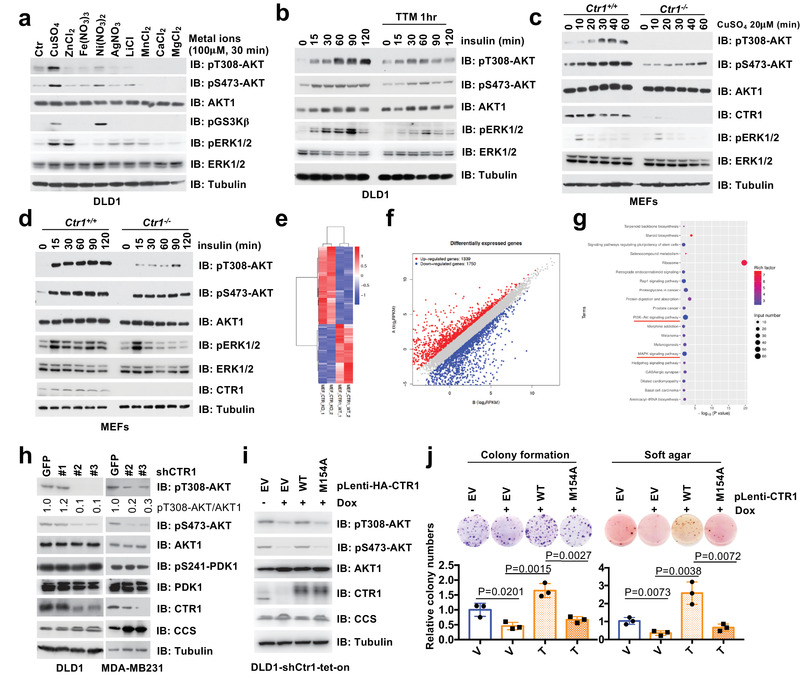
The CTR1‐copper signaling axis is important for AKT kinase activation and oncogenic function. a) HEK293 cells were serum‐starved for 12 h and treated with indicated metal ions (100 µm) for 30 min and harvested for immunoblot (IB) analysis. b) DLD1 cells were serum‐starved for 12 h, and subsequently treated with or without TTM (200 µm) for 1 h, and stimulated with insulin (0.1 µm) for indicated time points and harvested for IB analysis. c,d) *Ctr1^−/−^* and counterpart mouse embryonic fibroblasts (MEFs) were treated with copper (20 µm) or insulin (0.1 µm) in different time points before harvested for IB analysis. The RNAs derived from *Ctr1^−/−^* and counterpart MEFs were extracted and subjected to library construct and sequence. e) The different expression of RNAs derived from these cells were clustered, and f) the differentially expressed genes were blotted. g) The KEGG analyses were performed and the top 20 up‐regulated signal pathways in WT MEFs were illustrated. Among these pathways, PI3K‐AKT pathway and MAPK pathway were identified and underlined with red. h) MDA‐MB231 and DLD1 cells were infected with shRNAs against *CTR1* and *GFP*, and selected with puromycin (1 µg mL^−1^) for 72 h and harvested for IB analysis. The relative levels of pT308‐AKT were normalized with AKT1 by using the image J software. DLD1 cells infected with tet‐on inducible shRNAs against *CTR1* were selected with puromycin (1 µg mL^−1^) for 72 h, then infected with lentivirus encoding WT‐ or M154A‐CTR1, and selected with hygromycin (100 µg mL^−1^) for 5 days. Resulting cells were treated with doxycycline (1 µg mL^−1^) for 2 days and then i) subjected for IB analysis and j) colony formation assay (top panel). The relative colony numbers were normalized (bottom panel) (mean ± SD, *n* = 3; *t‐*test).

Given the important roles of metal ions in governing tumorigenesis and the frequent dysregulation of PI3K‐AKT signaling pathway in tumorigenesis,^[^ ^12^ ^]^ we assessed whether alterations of certain metal ions within the tumor microenvironment could influence AKT kinase activity. To this end, we challenged human embryonic kidney 293 (HEK293) and colon cancer DLD1 cell lines with a panel of metal ions, and observed that copper (Cu), but with a much lesser extent of the other ions we examined, including Zn^2+^(ZnCl_2_), Fe^3+^(Fe(NO_3_)_3_), Ni^2+^(Ni(NO_3_)_2_), Ag^+^(AgNO_3_), Li^+^(LiCl), Mn^2+^(MnCl_2_), Ca^2+^(CaCl_2_), and Mg^2+^(MgCl_2_), could significantly elevate AKT kinase activity indicated by the increase of AKT phosphorylation at Thr308 and Ser473, as well as its downstream substrate glycogen synthase kinase 3 beta (GSK3*β*) phosphorylation (**Figure** **1a**; Figure S1a, Supporting Information).

Next, we observed that copper could induce AKT activation in a dose‐dependent manner (Figure S1b–d, Supporting Information), which could be potently antagonized by copper chelators such as tetrathiomolybdate (TTM) and penicillamine (Figure 1b; Figure S1e–i, Supporting Information). Given that AKT phosphorylation in T308 by PDK1 contributes to around 70% AKT kinase activity while mTORC2‐mediated phosphorylation of pSer473‐AKT is a relatively minor contributor,^[^ ^16^, ^17^ ^]^ in the reminder of the study, we mainly chose to use pT308‐AKT as a faithful readout of AKT kinase activity. To explore the physiological role of copper in AKT activation, we observed that depleting the predominant copper transporter *Ctr1* in mouse embryonic fibroblasts (MEFs) could markedly decrease copper‐induced AKT phosphorylation (Figure 1c). Strikingly, depleting *Ctr1* also significantly diminished growth factor (such as insulin, epidermal growth factor (EGF), insulin‐like growth factor 1 (IGF), or platelet‐derived growth factor (PDGF))‐induced AKT phosphorylation compared with wild type (WT) MEFs (Figure 1d; Figure S2a–c, Supporting Information). In keeping with this finding, we performed ribonucleic acid (RNA) sequencing (RNA‐seq) assays, and discovered that, similar to the change of mitogen‐activated protein kinase (MAPK) signaling pathway, PI3K‐AKT signaling pathway was also dramatically induced under the condition of WT versus *Ctr1* depleting MEFs (Figure 1e–g; Table S1, Supporting Information). These results support the notion that the CTR1‐copper axis is an important physiological upstream regulator of the AKT kinase activity in cells. Furthermore, knockdown of *CTR1* could markedly attenuate AKT phosphorylation in cancer cells such as DLD1 and MDA‐MB231 (Figure 1h; Figure S2d,e, Supporting Information), resulting in decreased cellular colony formation and anchorage‐independent growth (Figure S2f–i,Supporting Information). To verify whether the oncogenic role of CTR1 is mediated by transporting copper, we introduced either WT or a noncopper binding mutant form of CTR1 (M154A)^[^ ^9^ ^]^ into *CTR1*‐depleted DLD1 or HEK293 cells, respectively. As a result, WT, but not M154A‐CTR1 could efficiently induce AKT phosphorylation (Figure 1i; Figure S2j, Supporting Information), and rescue the colony formation ability of *CTR1*‐depleted DLD1 and HEK293 cells (Figure 1j; Figure S2k, Supporting Information). These findings together suggest that the CTR1‐copper axis plays an important role in governing AKT kinase activity in cells.

**Figure 2:**
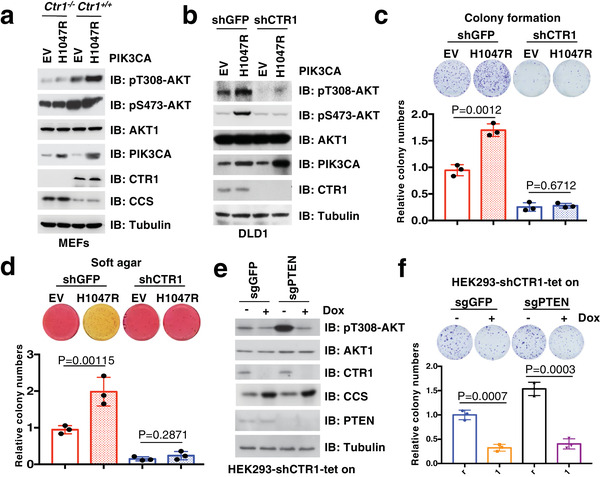
CTR1‐Copper axis displays an important role in AKT activation under pathological conditions. a,b) *Ctr1* deletion and counterpart MEFs or *CTR1* knockdown and control DLD1 cells were infected with lentivirus encoding PIK3CA‐H1047R, and selected with hygromycin (100 µg mL^−1^) for 5 days. Resulting cells were subjected for IB analysis. c,d) Cells generated in (b) were subjected to colony formation (c, top panel) and soft agar (d, top panel) assays. The relative colony numbers were normalized and plotted (c, d, bottom panels) (mean ± SD, *n* = 3; *t‐*test). e,f) HEK293 cells edited with CRISPR/Cas9 to generate *PTEN^−/−^* and control (sgGFP) cells were infected with tet‐on inducible shRNAs against *CTR1*, after selected with puromycin (1 µg mL^−1^) for 72 h, resulting cells were treated with doxycycline (1 µg mL^−1^) for 48 h and then subjected to IB analysis (e) and colony formation assays (f, top panel). The relative colony numbers were normalized and plotted (f, bottom panel) (mean ± SD, *n* = 3; *t‐*test).

It is well‐established that gain‐of‐function mutations of *PIK3CA* play critical roles for aberrant AKT activation in various human cancers.^[^ ^18^, ^19^ ^]^ Consistent with these reports, patient‐derived oncogenic mutant PIK3CA‐H1047R^[^ ^20^ ^]^ could markedly activate AKT in *Ctr1*‐proficient, but not in *Ctr1*‐deficient MEFs or *CTR1*‐depleted tumor cells (**Figure** **2a**,b). Biologically, PIK3CA‐H1047R‐induced cell proliferation and anchorage‐independent growth was significantly repressed by deleting *CTR1* in DLD1 cells (Figure 2c,d). Since *PTEN* is frequently mutated/deleted in human cancers and largely contributes to AKT activity,^[^ ^21^ ^]^ therefore, we employed a *PTEN*‐depleted cell line to study the potential roles of CTR1 in regulating AKT signaling pathway. We observed that *PTEN* deficiency‐induced AKT phosphorylation was markedly reduced by knocking down endogenous *CTR1* (Figure 2e), coupled with decreased cell capability of colony formation (Figure 2f). These findings implicate that the CTR1‐copper axis is likely dictating AKT activation and oncogenic functions under proto‐oncogenic conditions.

### 2.2 Copper Potentially Binds to PDK1

**Figure 3:**
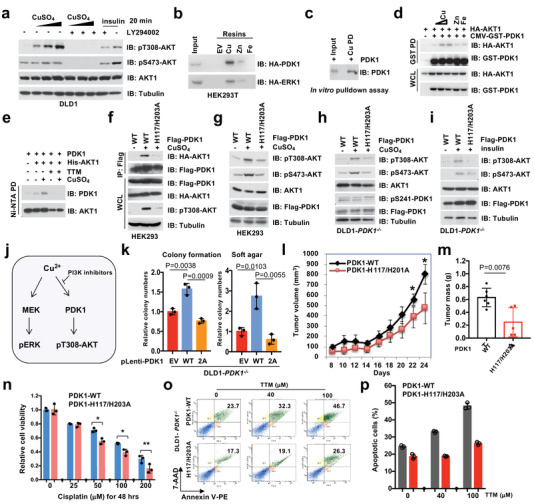
Copper potentially binds PDK1 to activate AKT kinase. a) DLD1 cells were serum‐starved for 12 h, and treated with or without LY294002 (10 µm) for 1 h before stimulated with copper (20, 50, and 100 µm) or insulin (0.1 µm) for 20 min. Resulting cells were harvested for IB analysis. b) NTA beads conjugated with Cu^2+^, Zn^2+^, and Fe^3+^ were used to perform pulldown (PD) assays with WCL derived from HEK293T cells transfected with indicated constructs. c) Cu‐PDC beads were used to perform the pulldown assays with recombinant GST‐PDK1 protein purified from bacteria, which has been cleaved by TEV protease before the PD assays. d) HEK293 cells were transfected with indicated constructs and treated with Cu^2+^, Zn^2+^, and Fe^3+^ after serum‐starvation for 12 h. Resulting cells were harvested and subjected for GST PD and IB analysis. e) In vitro binding assays were performed with insect cell purified PDK1 and His‐AKT1 proteins, in which copper (5 µm) and TTM (20 µm) were added as indicated. The Ni‐NTA beads were utilized for pulldown of His‐AKT1. The pulldown products were washed four times with NETN buffer (with 500 mm NaCl) and subjected for IB analysis. f,g) HEK293 cells transfected with indicated constructs or h,i) DLD1‐*PDK1^−/−^* cells infected with lentiviruses encoding indicated proteins were serum‐starved for 12 h and stimulated with copper (20 µm) or insulin (0.1 µm) before harvested for IP and IB analysis. j) A graphic illustration of the mechanistic model describing, similar to copper‐induced MEK‐ERK pathway, copper‐induced AKT‐pT308 by binding PDK1, which can be attenuated by PI3K inhibitors. k) Cells generated in (h) were subjected to colony formation and soft agar assays and the relative colony numbers were quantified (mean ± SD, *n* = 3; *t‐*test). Cells generated in (h) were subjected to xenograft assays. l) Tumor volumes were monitored (*t*‐test, *n* = 6) *p < 0.05, and m) tumor mass were weighted (mean ± SD, *n* = 6; *t*‐test). n) Cells generated in (h) were subjected to drug treatment assays, and the relative cell viability was plotted (mean ± SD, *n* = 3) *p < 0.05, **p < 0.01 (*t‐*test). o) Cells generated in (h) were treated with different doses of TTM and subjected to Annexin V/7‐AAD‐labeled apoptosis assays. p) Apoptotic cells were quantified (mean ± SD, *n* = 3).

Next, we sought to reveal the molecular mechanism underlying how copper activates AKT kinase in cells. In keeping with the notion that PI3K‐PDK1 is the primary upstream regulator of AKT‐pT308 and contributes to the majority activation of AKT kinase,^[^ ^22^ ^]^ PI3K inhibitors could dramatically reduce copper‐induced pT308‐AKT in different cell lines (**Figure** **3a**; Figure S3a, Supporting Information). Interestingly, copper‐induced pT308‐AKT was largely abolished by depleting *PDK1* (Figure S3b, Supporting Information), indicating that the activation of AKT by copper is likely dependent on the canonical PI3K‐PDK1 signaling pathway. Notably, we also observed that copper treatment could markedly induce AKT kinase activity in cells by performing in vitro kinase assays (Figure S3c, Supporting Information). Inspired by recent reports that copper could directly bind MEK to promote MEK interaction with and activation of its downstream targets extracellular signal‐regulated kinase 1 and 2 (ERK1/2),^[^ ^9^, ^23^ ^]^ we sought to explore whether copper plays a similar role to activate AKT kinase by binding PDK1. To this end, we found that copper enhanced the binding of PDK1 with AKT (Figure S3d, Supporting Information). Meanwhile, similar to MEK, PDK1 could be readily pulled down from cell lysates by copper‐resins, but not the resins conjugated with Zn^2+^, Fe^3+^, Co^2+^, or Mn^2+^ ions (Figure 3b; Figure S3e, Supporting Information). Moreover, the binding of copper with PDK1 could not be abrogated by PI3K inhibitors, such as LY294002 and wortmannin (Figure S3f, Supporting Information), indicating that copper binds PDK1 independent of phosphatidylinositol (3,4,5)‐trisphosphate (PIP_3_)‐mediated PDK1 plasma membrane translocation or conformational changes.

To evaluate whether copper could bind PDK1, we performed in vitro copper pulldown assays with recombinant PDK1 protein purified from bacteria or insect cells, in which the tagged GST was cleaved to exclude nonspecific binding, and observed that copper could pull down PDK1 in vitro (Figure 3c; Figure S3g,h, Supporting Information). These data together support that PDK1 is a novel binding partner of copper. To assess the potential roles of copper on the PDK1‐AKT oncogenic signaling, we sought to reveal whether copper could enhance PDK1 kinase activity. Consistent with a previous report that PDK1 undergoes auto‐phosphorylation and activation that is independent of growth factor stimulation,^[^ ^24^ ^]^ we observed that copper had minimal effect on phosphorylation of PDK1 at Ser241 (Figure S3i, Supporting Information), an indicator of PDK1 kinase activity.^[^ ^25^ ^]^ Furthermore, depletion of *CTR1* only minimally influenced pS241‐PDK1 in cells (Figure 1h; Figure S2e, Supporting Information). In keeping with the notion that CTR1‐copper is a major physiological regulator of PDK1‐AKT signaling, we observed that the presence of copper ion, but not other metal ions, such as Zn^2+^, Fe^3+^, or Ca^2+^, could enhance PDK1 binding with AKT in cells (Figure 3d; Figure S3j,k, Supporting Information). In addition, in vitro binding assays with insect cell purified PDK1 and AKT1 demonstrated that copper, but not other ions we examined, could enhance the binding of PDK1 and AKT1, which could be antagonized by the copper chelator TTM (Figure 3e; Figure S3l, Supporting Information). These findings indicate that copper could possibly activate AKT via enhancing its physical interaction with PDK1.

Given that the biological function of PI3K is to generate PIP_3_ and promote AKT and PDK1 membrane translocation,^[^ ^22^ ^]^ we asked whether copper could enhance PDK1 or AKT membrane translocation to augment their interaction. To this end, we observed that copper only moderately increased PDK1, but not AKT, plasma membrane translocation (Figure S3m, Supporting Information). Notably, copper‐induced PDK1 and AKT interaction could be largely abrogated by PI3K inhibitors (Figure S3n, Supporting Information), indicating that the generation of PIP_3_ and subsequent membrane co‐translocation of AKT and PDK1 is likely necessary for copper‐enhancement of PDK1/AKT interaction. These results together suggest that copper, at least in part, could enhance the interaction between PDK1 and its substrates including AKT, resulting in AKT activation in a PI3K dependent manner and leading to downstream biological consequences. Interestingly, the interaction of PDK1 with other downstream substrates such as S6K1, PLK1, and PKC1 were also enhanced by copper administration (Figure S3o–q, Supporting Information), indicating that other substrates of PDK1 are also likely to mediate copper functions in tumorigenesis.

### 2.3 Copper Binds PDK1 Mainly at H117 and H203

Using various deletion constructs, we mapped the PDK1 kinase domain as the major copper interacting module (Figure S4a,b, Supporting Information). In order to further identify the exact binding residues of copper at PDK1, copper‐induced in vitro oxidative assays coupled with mass spectrometry (MS) analyses were performed as previously reported,^[^ ^26^ ^]^ and multiple potential copper‐binding residues (oxidization in histidine (H) and methionine (M) residues, although the cysteine (C) residue also has the potential capability to bind copper) on PDK1 were identified (Figure S4c,d, Supporting Information). To exclude non‐specific sites obtained from in vitro oxidative reactions, we mutated these identified potential copper‐binding residues individually and observed that mutations of H117A and H203A, and to a lesser extent, M134A and H197A, in the PDK1 kinase domain, impaired the binding of copper with PDK1 in cells (Figure S4e,f, Supporting Information). Importantly, PDK1 mutations in H117A and H203A could partially decrease AKT‐pT308 compared with WT‐PDK1 expressing cells in the treatment of copper (Figure S4g, Supporting Information). In addition, these two residues (H117 and H203) are distinct in other AGC kinase family members we examined (Figure S4d, Supporting Information). Specifically, double mutations of PDK1 (referred as H117/H203A) could dramatically decrease the interaction of copper with PDK1 in cells (Figure 3f), suggesting that residues of H117 and H203 at PDK1 kinase domain are likely the major copper binding sites. Meanwhile, compared with PDK1‐WT expressing in DLD1‐*PDK1^−/‐^* and HEK293 cells, re‐introducing the mutant form of PDK1‐H117/H203A significantly decreased AKT‐pT308 induced by copper (Figure 3g,h; Figure S4g, Supporting Information), or by upstream stimuli such as insulin or EGF (Figure 3i; Figure S4h–l, Supporting Information). However, in the experimental setting of in vitro kinase assay, we found that the copper binding‐deficient H117/H203A mutant form of PDK1 exhibited comparable ability as WT‐PDK1 in phosphorylating AKT‐T308 (Figure S4m,Supporting Information). These results coherently indicate that copper is important for activating AKT signaling pathway in part via binding to PDK1 in cells (Figure 3j).

As a hallmark of cancer proliferation and survival, the dysregulation of AKT kinase activity contributes to more than 50% of human cancers.^[^ ^27^ ^]^ Hence, physiological regulation of the AKT oncogenic signaling pathway has been extensively investigated.^[^ ^12^ ^]^ Consistent with the crucial role of copper in PDK1‐mediated AKT activity, PDK1‐H117/H203A expressing DLD1‐*PDK1^−/−^* cells displayed reduced proliferation and anchorage‐independent growth capability compared with WT‐PDK1 expressing cells (Figure 3k; Figure S5a,b, Supporting Information), and tumor growth in xenograft mouse models (Figure 3l,m; Figure S5c–e, Supporting Information). In addition, copper markedly promoted cell proliferation and colony formation in WT, but not PDK1‐H117/203A expressing DLD1‐*PDK1^−/−^* cells (Figure S5f–h, Supporting Information). Interestingly, PDK1‐H117/H203A expressing cells also exhibited relatively more chemo‐sensitivity to cisplatin or etoposide treatment compared with PDK1‐WT expressing cells (Figure 3n; Figure S5i, Supporting Information). In keeping with these findings, the copper chelator TTM markedly decreased cell colony formation and anti‐apoptosis capability in WT, but not in H117/H203A‐PDK1 expressing cells (Figure 3o,p; Figure S5j,k, Supporting Information). These findings together suggest that the binding of copper/PDK1 likely plays an important role for promoting AKT kinase activity and oncogenic functions (Figure S5l, Supporting Information).

### 2.4 CTR1‐Copper Exhibits Oncogenic Functions in Part via Activating AKT Kinase

**Figure 4:**
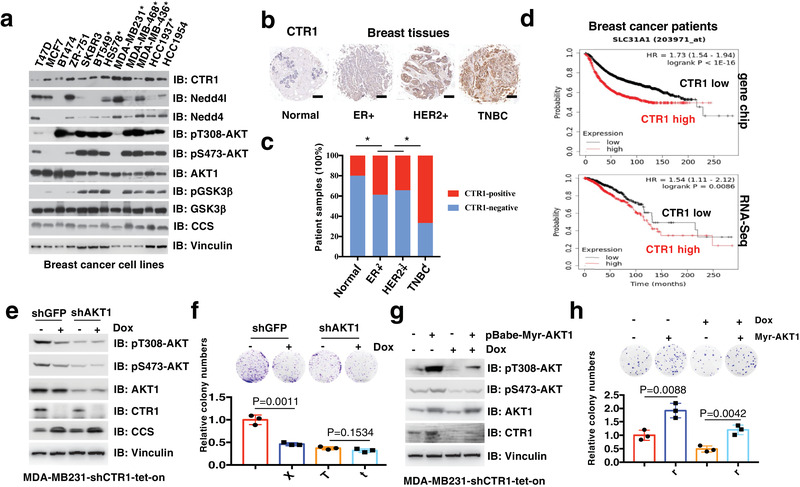
CTR1 is aberrantly expressed in breast cancer. a) IB analysis of WCL derived from a panel of breast cancer cell lines. Triple‐negative breast cancer cell lines were labeled with asterisk. The relative levels of CTR1 and pT308‐AKT were normalized with Vinculin or AKT1 respectively with the image J software. b) Image represented the CTR1 expression in different breast tissues detected with immunohistochemistry (IHC). c) The relative expression of CTR1 in different types of breast cancer were calculated and analyzed. *p < 0.05 (Chi‐square analysis). d) CTR1 expression in breast cancers derived from gene chip or RNA‐Seq database was analyzed and Kaplan–Meier plot for the survival of breast cancer patients was drawn via the website tool (http://kmplot.com/). Red curve indicates the high expression of CTR1, black curve indicates the low expression of CTR1 with the optimized cutoff. e,f) *AKT1* knockdown and WT MDA‐MB231 cells were infected with tet‐on inducible shRNAs against *CTR1*, and selected with puromycin (1 µg mL^−1^) for 5 days. Resulting cells were treated with doxycycline (1 µg mL^−1^) for 48 h and subject for IB analysis (e), colony formation assays (f, top panel). The relative colony numbers were normalized and plotted (f, bottom panel) (mean ± SD, *n* = 3; *t‐*test). g,h) Myr‐AKT1 stably expressing and control MDA‐MB231 cells were infected with tet‐on inducible shRNAs against *CTR1*, and selected with puromycin (1 µg mL^−1^) for 5 days. Resulting cells were treated with doxycycline (1 µg mL^−1^) for 48 h and subject for IB analysis (g), colony formation assays (h, top panel). The relative colony numbers were normalized and plotted (h, bottom panel) (mean ± SD, *n* = 3; *t‐*test).

Although our work revealed a potent role for copper in tumor malignancy by activating AKT signaling, the regulation of copper high‐affinity transporter CTR1, which confers more than 70% of copper uptake, remains largely elusive. Physiologically, CTR1 has been reported to highly express in renal tubules, ovary, and testis,^[^ ^28^ ^]^ but the mechanism of the abnormally elevated expression of CTR1 in human tumors is still unclear. Notably, we analyzed the expression of CTR1 in a panel of breast cancer cell lines and found that CTR1 was relatively high expression in triple‐negative breast cancer (TNBC) cell lines compared with estrogen receptor and progesterone receptor positive (ER^+^/PR^+^) or human epidermal growth factor receptor 2 positive (HER2^+^) cell lines (**Figure** **4a**). Moreover, CTR1 expression was further assessed in breast cancer tissues by immunohistochemistry (IHC) and demonstrated that CTR1 was highly expressed in breast tumor samples, especially in TNBCs, compared with adjacent normal breast tissues (Figure 4b,c). In addition, via a bioinformatic analysis, we found that relatively higher expression of CTR1 was associated with poor outcome of breast cancer patients (Figure 4d), indicating that CTR1 might serve as a potential prognosis marker for breast cancer.

Next, we sought to determine the potential role of AKT kinase in mediating the oncogenic function of CTR1. We observed that depletion of *CTR1* significantly decreased cell colony formation and soft agar growth in WT, but not in *AKT1* knockdown MDA‐MB231 cells (Figure 4e,f). Similar results were also observed in *AKT1/2* double knockout DLD1 cells (DLD1‐*AKT1/2^−/−^*) (Figure S6a,b, Supporting Information). More importantly, ectopic expression of the constitutively active AKT (myr‐AKT1) could antagonize *CTR1*‐deficiency‐induced repression of cellular colony formation and soft agar growth (Figure 4g,h). Meanwhile, knockdown of *CTR1* markedly inhibited AKT phosphorylation and cell malignant phenotype in WT, but not in H1171/H203A‐PDK1 expressing DLD1‐*PDK1^−/−^* cells (Figure S6c–e, Supporting Information). These findings suggest that CTR1 executes its oncogenic functions largely through copper‐mediated activation of the oncogenic AKT kinase.

### 2.5 Nedd4l Ubiquitinates and Subsequently Degrades CTR1

**Figure 5:**
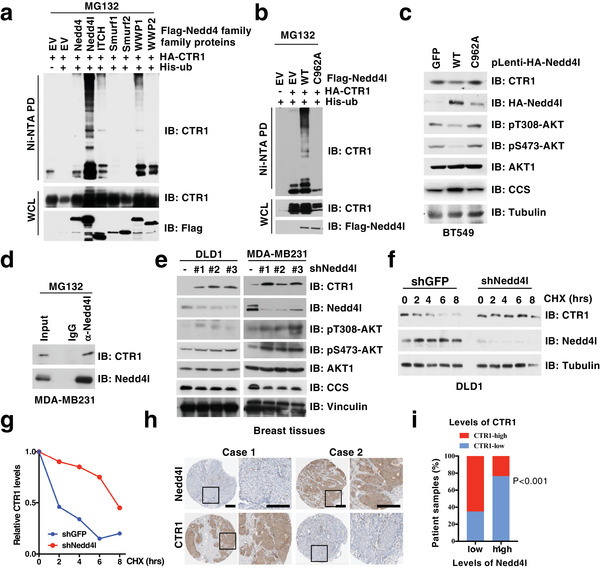
CTR1 is negatively regulated by Nedd4l‐mediated ubiquitination and subsequent degradation. a,b) IB analysis of in vivo ubiquitin products and WCL derived from HEK293T cells transfected with indicated constructs and treated with MG132 (10 µm) for 12 h before harvested. c) IB analysis of WCL derived from BT549 cells infected with indicated viruses and selected with puromycin (1 µg mL^−1^) for 3 days. d) IB analysis of IP products and WCL derived from MDA‐MB231 cells. IgG was used as a negative control. e) IB analysis of DLD1 and MDA‐MB231 cells infected with independent shRNAs against *NEDD4L*. f,g) IB analysis of WCL derived from DLD1 cells generated in (e) and treated with indicated time course of CHX (100 µg mL^−1^) (f). The relative protein levels were normalized and plotted (g). h) Image represents the CTR1 and Nedd4l expression in different breast tissues detected with immunohistochemistry (IHC). i) The association of CTR1 and Nedd4l expression in breast cancer tissues was analyzed (*p* < 0.001, Chi‐square analysis).

Although our work revealed a potential oncogenic role for CTR1, we found rare amplification or gain‐of‐function mutations of *CTR1* in human tumors via mining the cancer genome atlas (TCGA) datasets, indicating that the expression of CTR1 might be subjected to a transcriptional or post‐translational regulation. As such, it has been reported that *CTR1* gene expression is transcriptionally regulated by hypoxia induced factor (HIF) and Myc,^[^ ^29^, ^30^ ^]^ however, the control of CTR1 protein stability remains largely unclear. It is worthy note to mention that yeast CTR1 has been reported to be ubiquitinated and subsequently degraded by Rps5, the homologue of mammalian Neuronal precursor cell‐expressed developmentally downregulated 4 (Nedd4) family E3 ligase,^[^ ^31^ ^]^ which has evolved into nine family members in human beings.^[^ ^32^ ^]^ To further evaluate whether a specific member of the Nedd4 family E3 ligase controls CTR1 protein stability, we thus performed in vivo ubiquitination assays and observed that Nedd4l (also termed Nedd4‐2), but not other Nedd4 family E3 ligases, including Nedd4, Itchy E3 ubiquitin protein ligase (ITCH), SMAD specific E3 ubiquitin protein ligase 1 and 2 (Smurf1 and Smurf2) and WW domain protein E3 ubiquitin ligase 1 and 2 (WWP1 and WWP2), could induce the poly‐ubiquitination of CTR1 (**Figure** **5a**). Furthermore, we revealed that ectopic expression of Nedd4l, but not its enzyme‐inactive form Nedd4l‐C962A, could markedly induce CTR1 poly‐ubiquitination (Figure 5b). Meanwhile, ectopic expression of WT, but not Nedd4l‐C962A in BT549 cells, bearing relatively lower expression of endogenous Nedd4l, could down‐regulate CTR1 protein levels (Figure 5c). Further analyses revealed that Nedd4l could bind CTR1 at both exogenous and endogenous levels (Figure 5d; Figure S6f, Supporting Information).

To further understand the E3 functions of Nedd4l on CTR1 regulation, we observed that depletion of *NEDD4L* could markedly enhance endogenous CTR1 protein levels in multiple cell lines (Figure 5e; Figure S6g, Supporting Information). Moreover, depletion of *NEDD4L* prolonged the half‐life of CTR1 protein (Figure 5f,g). Clinically, we observed that the expression of Nedd4l was reduced in breast tumors compared to the adjacent normal tissues, meanwhile, the expression of Nedd4l was negatively correlated with CTR1 expression (Figure 5h,i). These findings together suggest that Nedd4l might be the physiological bona fide E3 ubiquitin ligase for CTR1.

### 2.6 Nedd4l Displays a Potent Tumor Suppressive Function by Suppressing CTR1‐AKT Signaling

**Figure 6:**
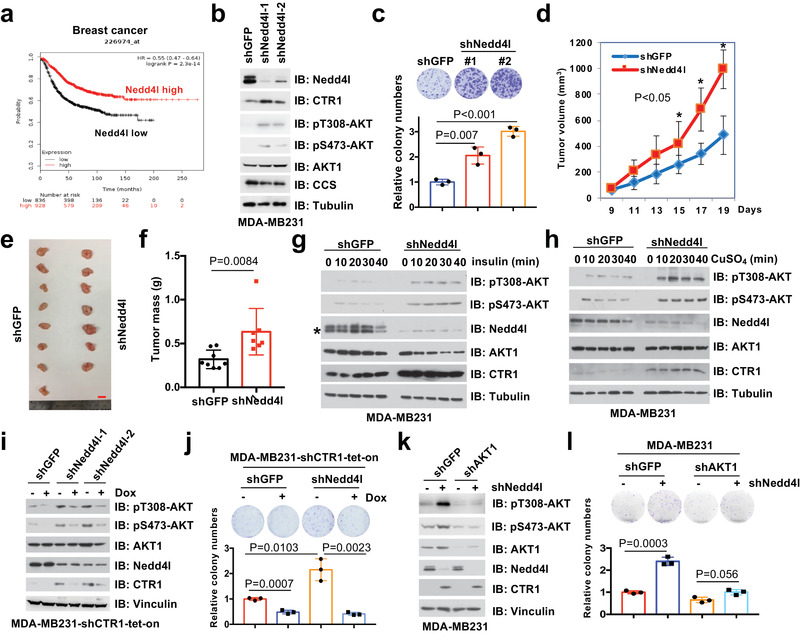
Nedd4l negatively regulates AKT kinase activity and executes tumor suppressor function largely by targeting CTR1 for ubiquitination‐mediated degradation. a) Nedd4l expression in breast cancer was analyzed and the Kaplan–Meier plot was drawn via a website tool (http://kmplot.com/). b,c) IB analysis of WCL derived from MDA‐MB231 cells infected with shRNAs against *NEDD4L* (b). Resulting cells were subjected for colony formation assays (c, top panel). Relative colony numbers were normalized and plotted (c, bottom panel) (mean ± SD, *n* = 3; *t‐*test). The cells generated in (b), were subjected to xenograft assays (5 × 10^6^ cells for injection). d) The mice were sacrificed once the tumors reached around 1.5 cm, and the tumor size was monitored (mean ± SD, *n* = 8; *p < 0.05, **p < 0.01, *t*‐test). The tumors were e) dissected and f) weighed (mean ± SD, *n* = 8; *t‐*test). Bar indicates 10 mm. MDA‐MB231 cells were infected with shRNAs against *NEDD4L* (shGFP was used as a negative control) and selected with puromycin (1 µg mL^−1^) for 72 h. Resulting cells were treated with g) insulin (0.1 µm) or h) copper (20 µm) in different time courses after serum‐starved for 12 h. Asterisk indicates the lower band as the major Nedd4l band. i,j) MDA‐MB231 cells were infected with shRNA against *NEDD4L*, selected with puromycin (1 µg mL^−1^) for 72 h, infected with tet‐on inducible shRNAs against *CTR1*, selected with puromycin (2 µg mL^−1^) for 5 days, and treated with or without doxycycline (1 µg mL^−1^) for 24 h before harvesting for IB analysis. Resulting cells generated from (i) were subjected to colony formation assays (j, top panel). The relative colony numbers were normalized and plotted (j, bottom panel) (mean ± SD, *n* = 3; *t‐*test). k,l) MDA‐MB231 cells were infected with shRNAs against *NEDD4L* and *AKT1* respectively, and selected with puromycin (1 µg mL^−1^) for 72 h for IB analysis (k). shGFP was used as a negative control. Resulting cells were subjected for colony formation assays (l, top panel). The relative colony numbers were normalized and plotted (l, bottom panel) (mean ± SD, *n* = 3; *t‐*test).

In support of Nedd4l as an upstream negative regulator of CTR1, data mining from the TCGA datasets indicates that genetic alterations in *NEDD4L*, such as deletion or mutations, occurred frequently in different type of tumors (Figure S6h, Supporting Information), and lower levels of *NEDD4L* were correlated with a poor outcome of breast cancer patients analyzed with the Kaplan–Meier plotter (http://kmplot.com/analysis/) (**Figure** **6a**).^[^ ^33^ ^]^ Although epithelial sodium channel (ENaC), Na^+^–Cl^−^ co‐transporter (NCC) and SMAD family member 2 and 3 (SMAD2/3) have been identified as Nedd4l downstream ubiquitination and degradation targets,^[^ ^34^, ^35^ ^]^ the physiological role of Nedd4l in tumorigenesis remains unclear. To this end, we observed that depletion of *NEDD4L* in cancer cells could markedly induce colony formation and anchorage‐independent growth in different cancer types (Figure 6b,c; Figure S6i,j, Supporting Information), and tumor growth in xenograft mouse models (Figure 6d–f; Figure S6k, Supporting Information).

Given the important role of the CTR1‐copper axis in activating AKT kinase, we further tested whether Nedd4l could regulate AKT kinase activity via negatively regulating the CTR1‐copper signaling. We observed that the mutations/deletion of *NEDD4L* was mutually exclusive with the activation of AKT signaling pathway (*PTEN* deletion/mutations, epidermal growth factor receptor *(EGFR*), *PI3KC*, or *AKT1* mutations/amplification) in breast cancers (Figure S6l, Supporting Information). In keeping with this finding, we examined a panel of breast cancer cell lines, and found that the expression of Nedd4l, but not Nedd4, a close member of Nedd4l, seemed like to negatively correlate with AKT phosphorylation, especially with AKT‐pT308 (Figure 4a).

**Figure 7:**
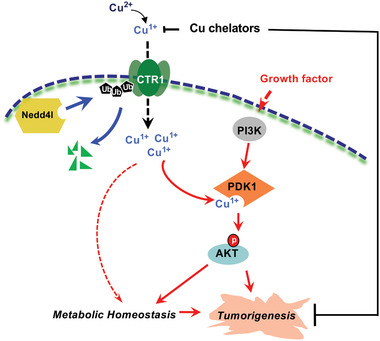
A proposed model for the important roles of the Nedd4l‐CTR1‐copper‐PDK1‐AKT signaling pathway in tumorigenesis.

More interestingly, re‐introducing WT, but not the enzyme‐inactive variant Nedd4l‐C962A, could inhibit AKT phosphorylation in *NEDD4L*‐depleted breast cancer cells, coupled with decreased CTR1 protein levels (Figure 5c). Furthermore, we found that depleting *NEDD4L* could enhance cellular response to insulin or copper‐induced AKT‐pT308 (Figure 6g,h), coupled with increased CTR1 protein levels (Figure 6g,h; Figure S7a–f, Supporting Information). Moreover, inducible knockdown of *CTR1* could markedly antagonize *NEDD4L* depletion‐induced AKT phosphorylation in different cell lines (Figure 6i; Figure S7g, Supporting Information), which was coupled with reduced colony formation in *NEDD4L*‐knockdown cells (Figure 6j; Figure S7h–k, Supporting Information). These results together indicate that stabilization of CTR1 is a major route through which *NEDD4L* depletion induces AKT kinase activity and oncogenic functions. In further support of the notion that the tumor suppressor function of Nedd4l is due to compromising AKT kinase activity, we found that depleting *NEDD4L*‐induced colony formation and anchorage‐independent growth could be largely abrogated in *AKT1*‐deficient MDA‐MB231 cells or *AKT1/2* double knockout DLD1 cells (Figure 6k,l; Figure S7l–n, Supporting Information). Given that the oncoprotein functions of CTR1 are mainly dependent on AKT activation (Figure 4f), our finding further demonstrates that the tumor suppressor function of Nedd4l is likely executed through governing the ubiquitination‐mediated degradation of CTR1 to dictate the downstream AKT signaling pathway. Hence, all these findings provide experimental evidence for a model whereby Nedd4l exerts its tumor suppressor roles largely by inhibiting the CTR1‐AKT signaling pathway in a copper‐PDK1 binding dependent manner (**Figure** **7**).

## 3 Discussion

The major physiological functions of copper have been associated with the metabolic regulations by governing the crucial metabolic enzyme activity, such as binding phosphodiesterase 3B (PDE3B) to amplify adenosine 3″,5″‐cyclic monophosphate (cAMP) signaling to regulate lipolysis,^[^ ^36^, ^37^ ^]^ or serving as an important player of ROS involving in neurodegenerative diseases, for example, Alzheimer's disease.^[^ ^38^ ^]^ Recently copper functions have been linked to tumorigenesis, especially to melanoma, thyroid or colon cancers by directly binding MEK to mediate v‐raf murine sarcoma viral oncogene homolog B1 (BRAF) valine 600 substituted by glutamic acid (BRAF^V600E^) oncogenic functions,^[^ ^9^, ^39^, ^40^ ^]^ or lung adenocarcinoma by enhancing autophagic ULK kinase functions.^[^ ^8^ ^]^ Here we report that copper can likely bind PDK1 to positively regulate AKT kinase activity via a PI3K dependent manner. However, although we have provided several pieces of experimental evidence including in cell and in vitro copper pulldown assays, in vitro oxidative assays and sites mutation binding assays to evaluate the critical role of copper in modulating PDK1‐AKT signaling activities, our data could not provide enough proof for proving the direct binding of copper to PDK1. Furthermore, whether copper could promote the oxidation of PDK1 or AKT to activate the PDK1‐AKT pathway needs to be further investigated. Moreover, the CTR1‐copper axis has been proven important for activating the PI3K‐PDK1‐AKT signaling pathway under both physiological and pathological conditions. Thus, in our studies, the copper chelators such as TTM have been validated to efficiently repress AKT kinase and with the potential to combat hyperactive AKT‐driven tumors. However, playing as a major metabolic mediator, copper also exhibits critical roles in regulating metabolic homeostasis, especially in regulating ROS, which recently has been implied in tumorigenesis.^[^ ^41^ ^]^ Therefore, aside from PDK1‐AKT, MEK‐ERK, and ULK‐autophagy signaling pathways, whether and how copper contributes to tumorigenesis by affecting metabolic homeostasis or other interacting partners warrant further investigation.

Copper transporters consist of two distinct paralogs, CTR1 and CTR2, and share distinct biological and biochemical features, in which CTR1 is considered the predominant transporter of copper with an extracellular copper binding domain.^[^ ^42^ ^]^ Cu^64^‐positron emission tomography scan (PET‐CT) has been recently developed to monitor tumor burden and metastasis in pre‐clinical studies due to the evidence of enriching copper in tumor regions,^[^ ^43^, ^44^ ^]^ which at least partially depends on the high expression of CTR1 in tumors.^[^ ^45^ ^]^ However, how CTR1 expression is abnormally elevated in tumors and whether it plays oncogenic function via copper‐mediated activation of oncogenic pathways is not well defined. In this study, we identified Nedd4l as one of the upstream physiological E3 ligases for CTR1, and depletion of *NEDD4L* could significantly enhance CTR1 protein levels and lead to tumor growth both in cells and xenografted mouse models. Mechanistically, the accumulation of copper could markedly activate AKT kinase via its potential interaction with AKT upstream kinase, PDK1. Notably, the presence of CTR1 and copper is important for PDK1 binding and activating AKT in both physiological and pathological conditions. Therefore, activation of the PDK1‐AKT signaling pathway is likely the major route through which the CTR1‐copper axis executes its oncogenic functions.

Nedd4l is the predominant E3 ligase responsible for ubiquitination and degradation of ENaC and NCC, thus, the Nedd4l protein in mice is essential for animal survival. As a result, the polymorphisms of NEDD4L are associated with human hypertension.^[^ ^34^, ^35^ ^]^ Nedd4l has also been shown to negatively regulate several oncogenic proteins, such as EGFR, transforming growth factor beta (TGF*β*) receptor and WNT signaling,^[^ ^46^, ^47^ ^]^ however, the tumor suppressive roles of Nedd4l is not well defined yet. Here, we demonstrated a novel substrate of Nedd4l, CTR1, through which Nedd4l could negatively regulate AKT kinase activity and oncogenic functions. In echoing this finding, depletion of either *CTR1* or *AKT1* both could compromise Nedd4l tumor suppressor roles in repressing cancer cell malignant phenotypes. However, to further validate the tumor suppressor function of Nedd4l, the *Nedd4l* conditional knockout mice, particularly in lung tissues will be worth to generate and crossed with lung conditional *Kras^G12D^* mice,^[^ ^48^ ^]^ to monitor the lung tumorigenesis, which would provide robust genetic evidence to elucidate whether Nedd4l plays critical roles in tumorigenesis by targeting CTR1‐AKT signaling pathway. Moreover, except Nedd4l‐mediated degradation of CTR1, whether other E3 ligases could contribute to CTR1 protein levels warrant to be further tested.

As the evidence of CTR1 in prediction of platinum‐based chemotherapeutics seems more controversial,^[^ ^49^, ^50^, ^51^ ^]^ here we show that CTR1 plays an important role in positively regulating AKT kinase activity, which has been considered as one of the most important causes of cancer cell resistant to platinum‐based treatment.^[^ ^52^ ^]^ More interestingly, in combination with copper chelator TTM, the efficiency of chemo‐drugs could be largely enhanced possibly due to the decrease of copper‐induced AKT activity and its oncogenic functions. However, targeting PI3K‐AKT signaling has been largely limited in clinic for cancer therapies in part due to the essential roles of AKT in physiological processes.^[^ ^53^, ^54^ ^]^ By contrast, the first PIK3CA inhibitor, Ipelisib recently has been approved by FDA. This drug displays a promising potential on target advanced or metastatic breast cancers bearing a mutation in the *PIK3CA* gene, which largely encourages the field to further investigate the upstream regulation of AKT signaling. Thus, our findings that high expression of CTR1 could activate AKT signaling will provide another potential option for combating hyper‐active AKT‐driven cancers.

## 4 Conclusion

Overall, in this study, we not only reveal Nedd4l‐mediated ubiquitination as an upstream negative regulator of CTR1, but also identify a downstream effector of copper by potentially binding PDK1 to activate AKT oncogenic signaling pathway. Importantly, these findings highlight the potential therapeutic strategy by targeting the CTR1‐copper oncogenic axis for hyperactive AKT‐driven cancers, especially in the setting of breast cancer.

## 5 Experimental Section

### Cell Culture and Transfection or Infection

HEK293, HEK293T cells were cultured in Gibco Dulbecco's Modified Eagle Medium (DMEM) supplemented with 10% fetal bovine serum (FBS), penicillin (100 units), and streptomycin (100 µg mL^−1^). DLD1‐*AKT1^−/−^AKT2^−/−^* (termed *AKT1/2* ^−/−^), DLD1‐*PDK1^−/−^*, and wild‐type DLD1 cells were kindly provided by Dr. Bert Vogelstein (Johns Hopkins University School of Medicine). *Ctr1^−/−^* and counterpart MEFs (gifts from Dr. Chris Counter, Duke University Medical Center) were maintained in DMEM medium supplemented with 10% FBS. Breast cancer cell lines including T47D, MCF7, BT474, ZR‐751, SKBR3, BT549, HS578, MDA‐MB231, MDA‐MB468, MDA‐MB436, HCC1937, HCC1954 (gift from Dr. Piotr Sicinski, Danna Farber Cancer Center, Harvard Medical School) were cultured in RPMI 1640 medium or McCoy's 5A medium supplemented with 10% FBS. sgAKT1 and sgPTEN HEK293 cell lines were generated via a clustered regularly interspaced short palindromic repeats (CRISPR) and CRISPR‐associated protein 9 (Cas9) methods as described before.^[^ ^55^ ^]^ Cell transfection was performed using Lipofectamine and Plus reagents, as described previously.^[^ ^14^ ^]^ Packaging of lentiviral short hairpin RNA (shRNA) or complementary DNA (cDNA) expressing viruses and retroviral cDNA expressing viruses, as well as subsequent infection of various cell lines were performed according to the protocols described previously.^[^ ^14^ ^]^ Following viral infection, cells were maintained in the presence of hygromycin (200 µg mL^−1^) or puromycin (1 µg mL^−1^), depending on the viral vectors used to infect cells.

### Antibodies and Reagents

All antibodies were used at a 1:1000 dilution in TBST buffer with 5% non‐fat milk for western blot. Anti‐CTR1 antibody (13086), anti‐phospho‐Ser473‐AKT antibody (4060), anti‐phospho‐Thr308‐AKT antibody (2965), anti‐AKT1 antibody (2938), anti‐AKT total antibody (4691), anti‐phospho‐Ser9‐GSK3*β* antibody (5558), anti‐GSK3*β* antibody (12456), anti‐phospho‐ERK1/2 (4307), anti‐ERK1/2 (4695), anti‐Nedd4l (4013) antibody, anti‐myc (2276) antibody, anti‐AIF (5318) antibody, anti‐PIK3CA (4249) antibody, anti‐GST antibody (2625), anti‐pS241‐PDK1 antibody (3438) were obtained from Cell Signaling Technology. Polyclonal anti‐HA antibody (sc‐805) and PDK1 antibody (sc‐17765) were obtained from Santa Cruz. Polyclonal anti‐Flag antibody (F‐2425), monoclonal anti‐Flag antibody (F‐3165, clone M2), anti‐Tubulin antibody (T‐5168), anti‐Flag agarose beads (A‐2220), anti‐HA agarose beads (A‐2095), peroxidase‐conjugated anti‐mouse secondary antibody (A‐4416), and peroxidase‐conjugated anti‐rabbit secondary antibody (A‐4914) were obtained from Sigma. Monoclonal anti‐HA antibody (MMS‐101P) was obtained from Covance.

### DNA Constructs and Mutagenesis

Constructs of pcDNA3‐HA‐AKT1, pcDNA3‐HA‐AKT2, pcDNA3‐HA‐AKT3, pcDNA3‐HA‐myr‐AKT1, pcDNA3‐HA‐AKT1‐E17K, pcDNA3‐myc‐PDK1, pCMV‐Flag‐PDK1 were previously described.^[^ ^14^ ^]^ pcDNA3‐Flag‐Nedd4, pcDNA3‐Flag‐Nedd4l, pcDNA3‐Flag‐itch, pcDNA3‐Flag‐Smurf1, pcDNA3‐Flag‐Smurf2, pcDNA3‐Flag‐WWP1, pcDNA3‐Flag‐WWP2 were previously described.^[^ ^56^ ^]^ pcDNA3‐HA‐S6K1, pcDNA3‐HA‐RSK1, pcDNA3‐HA‐PLK1 were purchased from Addgene. pLenti‐PIK3CA‐H1047R were obtained from Dr. Alex Toker group. pCMV‐GST‐PDK1, pCMV‐GST‐PDK1‐N, pCMV‐GST‐PDK1‐KD, pCMV‐GST‐PDK1‐Poly‐S, pCMV‐GST‐PDK1‐PH, pCMV‐GST‐CTR1, pCMV‐GST‐AKT1, pLenti‐HA‐Nedd4l, pLenti‐PDK1 and pGEX‐4T‐PDK1 were cloned into indicated vectors. Details of plasmid constructions are available upon request. shRNAs against *CTR1*, *NEDD4L* were purchased from Sigma. The tet‐on inducible shRNA‐CTR1 were generated with pLKO‐tet‐on constructs and primers:

hCTR1‐tet‐on‐sh1‐Foward,

5′CCGGGCGTAAGTCACAAGTCAGCATCTCGAGATGCTGACTTGTGACTTACGCTTTTT;

hCTR1‐tet‐on‐sh1‐Reverse,

5′AATTAAAAAGCGTAAGTCACAAGTCAGCATCTCGAGATGCTGACTTGTGACTTACGC;

hCTR1‐tet‐on‐sh2‐Foward,

5′CCGGCGGTACAGGATACTTCCTCTTCTCGAGAAGAGGAAGTATCCTGTACCGTTTTT;

hCTR1‐tet‐on‐sh2‐Reverse,

5′AATTAAAAACGGTACAGGATACTTCCTCTTCTCGAGAAGAGGAAGTATCCTGTACCG.

Various PDK1, Nedd4l and CTR1 mutants were generated using the QuikChange XL Site‐Directed Mutagenesis Kit (StrataGene) according to the manufacturer's instructions.

### Cell Fractionations

Cell fractionations were performed with Cell Fractionation Kit (CST9038). Kinase inhibitors Wortmannin (Selleck S2758), and LY294002 (Selleck S1105) were used at the indicated doses. Etoposide (Sigma, E1383) and doxorubicin (Selleck, S1208), insulin (Invitrogen 41400‐045), EGF (Sigma E9644), PDGF (Sigma SRP3123) and IGF (Sigma SRP3069) were used at the indicated doses. MG132 (Enzo Life Science, BML‐PI102) was used at the indicated doses. TTM and penicillamine were purchased from Sigma. Metals including CuSO_4_, ZnCl_2_, Fe(NO_3_)_3_, Ni(NO_3_)_2_, AgNO_3_, LiCl, MnCl_2_, CaCl_2_, and MgCl_2_ were purchased from Fisher Scientific. Copper‐PDC beads were purchased from Affiland. Metal resin was generated with the metal ions with Glutathione‐Sepharose slurry (Pierce) or NTA Agarose (Qiagen 30310) for 1 h in room temperature and washed twice with phosphate buffered saline (PBS) buffer.

### Immunoblot (IB) and Immunoprecipitation (IP) Analyses

Cells were lysed in EBC lysis buffer (50 mm Tris pH 7.5, 120 mm NaCl, 0.5% NP‐40) supplemented with protease inhibitors (Complete Mini, Roche) and phosphatase inhibitors (phosphatase inhibitor cocktail set I and II, Calbiochem). The protein concentrations of whole cell lysates were measured by the Beckman Coulter DU‐800 spectrophotometer using the Bio‐Rad protein assay reagent. Equal amounts of whole cell lysates were resolved by SDS‐PAGE and immunoblotted with indicated antibodies. For immunoprecipitation analysis, 1000 µg lysates were incubated with the indicated antibody (1–2 µg) for 3–4 h at 4 °C followed by 1 h incubation with Protein A/G Sepharose beads (GE Healthcare). The recovered immuno‐complexes were washed five times with NETN buffer (20 mm Tris, pH 8.0, 150 mm NaCl, 1 mm EDTA, and 0.5% NP‐40) before being resolved by SDS‐PAGE and immunoblotted with indicated antibodies. Quantification of the immunoblot band intensity was performed with Image J software.

### In Vivo Ubiquitination Assay

His‐ubiquitin along with PDK1 and different members of Nedd4 were transfected into HEK293T cells. Thirty‐six hours after transfection, cells were treated with 10 µm carbobenzoxy‐Leu‐Leu‐leucinal (MG132) for 12 h and washed with PBS twice, and then were lysed in buffer A (6 m guanidine‐HCl, 0.1 m Na_2_HPO_4_/NaH_2_PO_4_, and 10 mm imidazole (pH 8.0)) and subjected to sonicate. After high‐speed centrifuged, the supernatants were incubated with nickel‐beads (Ni‐NTA) (Qiagen) for 3 h at room temperature. The products were washed twice with buffer A, twice with buffer A/TI (1 volume buffer A and 3 volumes buffer TI), and one time with buffer TI (25 mm Tris‐HCl and 20 mm imidazole (pH 6. 8)). The pull‐down proteins were resolved in 8% SDS‐PAGE for immunoblot analysis.

### Breast Tumor Microarrays (TMA) and IHC Staining

Tissue array (BC081120d) containing 10 cases of adjacent normal breast tissues, 100 cases of invasive ductal carcinoma was obtained from Biomax (including ER^+^ or PR^+^, 63; HER2^+^, 32; TNBC, 21). Immunohistochemistry was performed on four micron‐thick, formalin‐fixed paraffin‐embedded (FFPE) sections using an anti‐CTR1 polyclonal antibody (Novus NB100‐402, 1:200) and anti‐Nedd4l polyclonal antibody (Sigma, HPA024618, 1:200). FFPE sections were deparaffinized using xylene and rehydrated in graded ethanol. Sections were heated with a pressure cooker to 125 °C for 30 s and 90 °C for 10 s in citrate buffer (pH 6.0) for antigen retrieval. All sections were incubated with peroxidase (Dako #S2003) and protein blocking reagents (Dako #X0909) for 5 min each. Sections were then incubated with anti‐CTR1 antibody and anti‐Nedd4l antibody diluted in Dako diluent with background reducing components (Dako #S3022) for 1 h at room temperature. Following primary antibody incubation, sections were incubated with monoclonal mouse anti‐rabbit immunoglobulins (Dako #M0737) for 30 min at room temperature. Afterward, sections were incubated with Envision+ System‐HRP Labeled Polymer Anti‐Rabbit (Dako #K4003) for 30 min. All sections were developed using the DAB chromogen kit (Dako #K3468) and lightly counterstained with hematoxylin.

### Annexin‐V/Amino Actinomycin D Double Staining Assay

To detect cellular apoptosis, cells treated with the indicated concentration of TTM, were co‐stained with Annexin‐V‐phycoerythrin (PE) and amino actinomycin D (7‐AAD) from Annexin V‐PE apoptosis detection kit I (BD Biosciences) according to the manufacturer's instructions. Stained cells were measured with fluorescence‐activated cell sorting (FACS).

### Purification of Glutathione S‐Transferase (GST)‐Tagged Proteins from Bacteria

Recombinant GST‐conjugated PDK1 or AKT1 fragments were generated by transforming the BL21 (DE3) *Escherichia coli* strain. Starter cultures (5 mL) grown overnight at 37 °C were inoculated (1%) into larger volumes (500 mL). Cultures were grown at 37 °C until an O.D. of 0.8, following which protein expression was induced for 12–16 h using 0.1 mm isopropyl *β*‐d‐1‐thiogalactopyranoside (IPTG) at 16 °C with vigorous shaking. Recombinant proteins were purified from harvested pellets. Pellets were re‐suspended in 5 mL EBC buffer and sonicated (5 cycles of 10 s each at 50% output). Insoluble proteins and cell debris were discarded following centrifugation in a table‐top centrifuge (13 000 rpm/4 °C/15 min). Each 1 mL supernatant was incubated with 50 µL of 50% Glutathione‐Sepharose slurry (Pierce) or Ni‐beads (Qiagen) for 3 h at 4 °C. The Glutathione beads were washed three times with PBS buffer (1 mL per wash) and stored at 4 °C in PBS buffer containing 10% glycerol or eluted by elution buffer. The Ni‐beads were washed three times with 50 mm Tris‐Cl (pH 8.0) containing 20 mm Imidazole, and eluted by Tris‐buffers containing 100 mm Imidazole. Recovery and yield of the desired proteins (or complexes) was confirmed by analyzing 10 µL of beads by coomassie blue staining and quantified against BSA standards.

### In Vitro Kinase Assay

AKT in vitro kinase assays were modified from a protocol described previously.^[^ ^14^ ^]^ Briefly, 1 µg of the bacterially purified GST‐GSK3*β* fusion proteins were incubated with immunoprecipitated HA‐AKT1 from serum‐deprived HEK293 after treated with or without 20 µm Cu in the kinase buffer (200 µm ATP, 50 mm Tris pH 7.5, 1 µm MnCl_2_, 2 mm DTT, 1 mm EGTA) for 30 min at 30 °C. The reaction was subsequently stopped by the addition of 3 × SDS loading buffer and resolved by SDS‐PAGE. Phosphorylation of GST‐GSK3*β* was detected by pS9‐GSK3*β* antibody. PDK1 in vitro kinase assays were performed with the similar protocol as done in AKT in vitro kinase assay. Briefly, immunoprecipitated Flag‐WT‐ or H117/H203A‐PDK1 from HEK293 cells were used as the source of kinases and insect cell purified His‐AKT1 as the substrate for kinase assays. Phosphorylation of His‐AKT1 was detected by pT308‐AKT antibody.

### Analysis of In Vitro Oxidative Results by Liquid Chromatography‐Tandem Mass Spectrometry (LC/MS‐MS)

For mass spectrometry analysis, bacterially purified GST‐PDK1 was used for in vitro oxidative assays in the presence/absence of copper, and the resulting products were subjected for mass spectrometry analysis. The band containing PDK1 was reduced with 10 mm DTT for 30 min, alkylated with 55 mm iodoacetamide for 45 min, and in‐gel‐digested with trypsin enzymes. The resulting peptides were extracted from the gel and analyzed by microcapillary reversed‐phase (C_18_) LC‐MS/MS), using a high resolution QExactive HF Orbitrap (Thermo Fisher Scientific) in positive ion DDA mode (Top 8) via higher energy collisional dissociation (HCD) coupled to a Proxeon EASY‐nLc II nano‐HPLC.^[^ ^57^ ^]^ MS/MS data were searched against the Uniprot Human protein database (version 20151209 containing 21 024 entries) using Mascot 2.5.1 (Matrix Science) and data analysis was performed using the Scaffold 4.4.8 software (Proteome Software). Peptides and modified peptides were accepted if they passed a 1% FDR threshold.

### RNA Sequencing (RNA‐seq)

UID RNA‐seq experiment and high through‐put sequencing and data analysis were conducted by Seqhe alth Technology Co., Ltd. (Wuhan, China). Briefly, Total RNAs were extracted by using TRIzol Reagent (Invitrogen, cat. NO 15596026). DNA digestion was carried out after RNA extraction by DNaseI. RNA quality was determined by examining A260/A280 and confirmed by 1.5% agarose gel electrophoresis. 2 µg total RNAs were used for stranded RNA sequencing library preparation using KC‐Digital Stranded mRNA Library Prep Kit for Illumina (Catalog NO. DR08502, Wuhan Seqhealth Co., Ltd. China) following the manufacturer's instruction. The kit eliminates duplication bias in PCR and sequencing steps, by using unique molecular identifier (UMI) of 8 random bases to label the pre‐amplified cDNA molecules. The library products corresponding to 200–500 bps were enriched, quantified and finally sequenced on Novaseq 6000 sequencer (Illumina) with PE150 model. The raw sequencing data was first filtered by Trimmomatic (version 0.36), low‐quality reads were discarded. Clean Reads were further treated with in‐house scripts to eliminate duplication bias introduced in library preparation and sequencing. After all sub‐clusters were generated, multiple sequence alignment was performed to get one consensus sequence for each sub‐cluster. After that, the de‐duplicated consensus sequences were used for standard RNA‐seq analysis. They were mapped to the reference genome of mouse using STAR software (version 2.5.3a) with default parameters. A *p*‐value cutoff of 0.05 and fold‐change cutoff of 2 were used to judge the statistical significance of gene expression differences. Kyoto encyclopedia of genes and genomes (KEGG) enrichment analysis for differentially expressed genes were both implemented by KOBAS software (version: 2.1.1) with a *p*‐value cutoff of 0.05 to judge statistically significant enrichment.

### Colony Formation Assays

Cells were seeded into 6‐well plates (300 or 600 cells per well) and left for 8–16 days until formation of visible colonies. Colonies were washed with PBS and fixed with 10% acetic acid/10% methanol for 20 min, then stained with 0.4% crystal violet in 20% ethanol for 20 min. After staining, the plates were washed and air‐dried, and colony numbers were counted. Three independent experiments were performed to generate the standard error of the difference (SED).

### Soft Agar Assays

The anchorage‐independent cell growth assays were performed as described previously.^[^ ^14^ ^]^ Briefly, the assays were performed using 6‐well plates where the solid medium consists of two layers. The bottom layer contains 0.8% noble agar and the top layer contains 0.4% agar suspended with 1 × 10^4^ or 3 × 10^4^ cells. 500 µL complete DMEM medium was added every 7 days to keep the top layer moisture and 4 weeks later the cells were stained with iodonitrotetrazolium chloride (1 mg mL^−1^) (sigma I10406) for colony visualization and counting. Three independent experiments were performed to generate the standard deviation (SD) .

### Mice and Ethics Statement

Mouse xenograft assays were performed as described previously.^[^ ^58^ ^]^ Briefly, 2 × 10^6^ DLD1‐*PDK1^−/−^* cells stably expressing WT or H117/H203A mutant form of PDK1, or 5 × 10^6^ MDA‐MB231 cells were injected into the flank of 8 female nude mice (NCRNU‐M‐M from Taconic, 4–5 weeks of age). Tumor size was measured every three days with a caliper, and the tumor volume was determined with the formula: *L* × *W* ^2^ × 0.52, where *L* is the longest diameter and *W* is the shortest diameter. All experimental procedures were approved by the Institutional Animal Care & Use Committee (IACUC, RN150D) at Beth Israel Deaconess Medical Center with protocol #043‐2015. The research projects that are approved by the IACUC were operated according to the applicable Institutional regulations. The Institute is committed to the highest ethical standards of care for animals used for the purpose of continued progress in the field of human cancer research.

### Statistical Analysis

Statistical analyses were performed using GraphPad 8.0 (GraphPad Software Inc.). For all experiments, unless otherwise indicated, *n* = 3. Data were shown as means ± standard deviation (SD), and analyzed using two‐tailed Student's* t*‐test and Chi‐Square test. Values of *p* < 0.05 were considered statistically significant.

## Conflict of Interest

W.W. and P.P.P. are co‐founder and stockholder of the Rekindel Therapeutics. All other authors declare no conflict of interest.

## Supporting information

Supporting Information

Supplemental Table 1

## Data Availability

The data that support the findings of this study are available from the corresponding author upon reasonable request.
