# Supplementary material for: Copper Promotes Tumorigenesis by Activating the PDK1‐AKT Oncogenic Pathway in a Copper Transporter 1 Dependent Manner
Source: Adv Sci (Weinh). 2021 Jul 18;8(18):2004303. doi: 10.1002/advs.202004303 (PMC8456201; doi:10.1002/advs.202004303)

© 2021 Wiley-VCH GmbH

Supporting Information

for *Adv. Sci.,* DOI: 10.1002/advs.202004303

Copper Promotes Tumorigenesis by Activating the PDK1-AKT Oncogenic Pathway in a Copper Transporter 1 Dependent manner

Jianping Guo, Ji Cheng Nana Zheng, Xiaomei Zhang, Xiaoming Dai, Linli Zhang, Changjiang Hu, Xueji Wu, Qiwei Jiang, Depei Wu, Hitoshi Okada, Pier Paolo Pandolfi, Wenyi Wei**

Supporting Information

Copper promotes tumorigenesis by activating the PDK1-AKT oncogenic pathway in a copper transporter 1 dependent manner

Jianping Guo,[1,2,8,] Ji Cheng[1,3,8] Nana Zheng,[4,8] Xiaomei Zhang,[2] Xiaoming Dai,[1] Linli Zhang,[5] Changjiang Hu,[1] Xueji Wu,[2] Qiwei Jiang,[2] Depei Wu,[4] Hitoshi Okada,[6] Pier Paolo Pandolfi,[7] Wenyi Wei[1,*]*

Author contributions

This study was conceived and designed by J.Guo, W.Wei; Development of methodology: J.Guo, J.Cheng, X.Zhang, L.Zhang, W.Wei; Acquisition of data (provided animals, acquired and managed patients, provided facilities, ect.): J.Guo, J.Cheng, N.Zheng, X.Zhang, L.Zhang, C.Hu, X.Wu; Analysis and interpretation of data (e.g., statistical analysis, biostatistics, computational analysis): J.Guo, J.Cheng, X.Zhang, C.Hu, Q.Jiang and W.Wei; Writing, review, and/or revision of the manuscript: J.Guo, X.Zhang, X.Zhang, C.Hu, X.Dai, X.Wu, Q.Jiang and W.Wei; Administrative, technical, or material support (i.e., reporting or organizing data, constructing databases): N.Zheng, C.Hu, X.Wu, D.Wu, H.Okada, P.P.Pandolfi,; Study supervision: J.Guo, W.Wei. Approved manuscript: all authors.

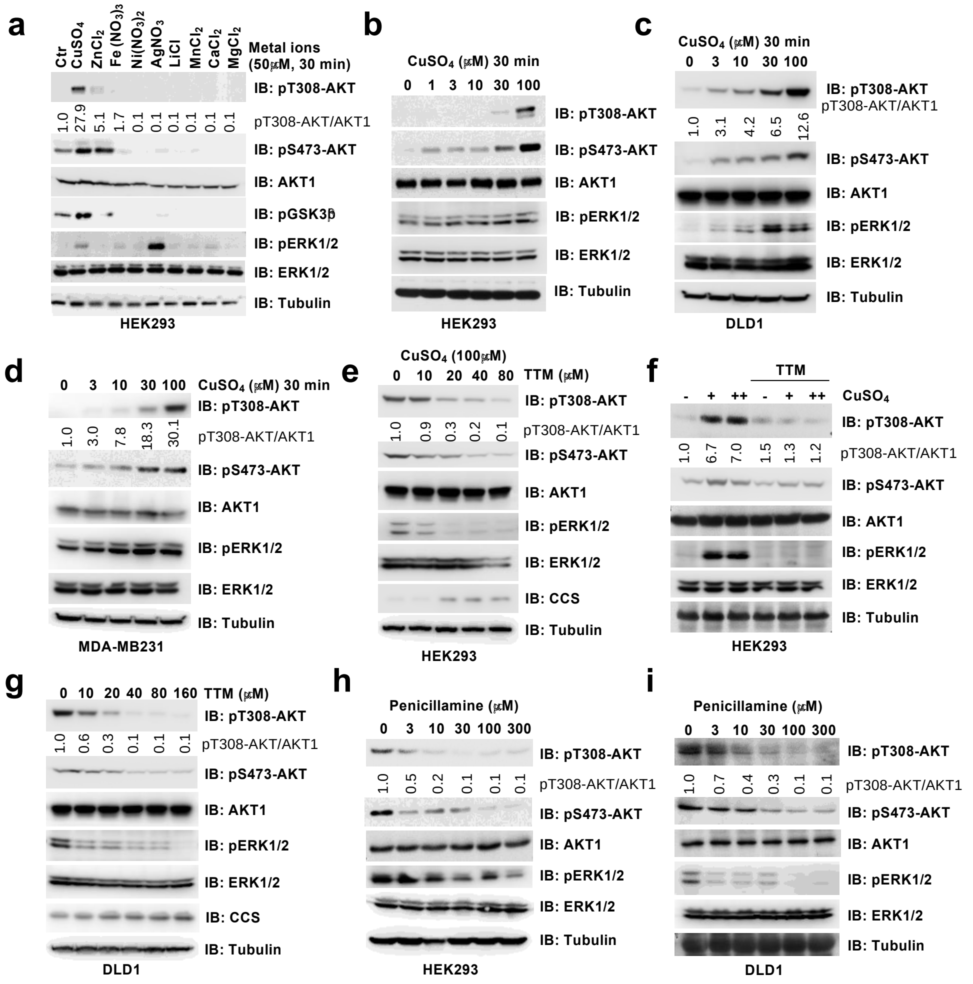

Figure S1. Copper activates the AKT kinase. a) DLD1 cells were serum-starved for 12 hrs and treated with indicated metal ions (50 μM) for 30 mins before harvested for immunoblot (IB) analysis. Where indicated, the relative levels of pT308-AKT signal in relative to AKT1 were normalized by using the image J software. b-d) HEK293 (b), DLD1 (c) and MDA-MB231 (d) cells were serum-starved for 12 hrs and treated with indicated concentration of copper for 30 minutes. e,f) HEK293 cells were serum-starved for 12 hrs and stimulated with or without copper before treated with or without different concentration of TTM for 2 hrs. g) IB analysis of WCL derived from DLD1 cells cultured in medium treated with different dose of TTM for 20 minutes. h,i) HEK293 (h) or DLD1 (i) cells treated with different doses of penicillamine for 3 hrs, and harvested for IB analysis.

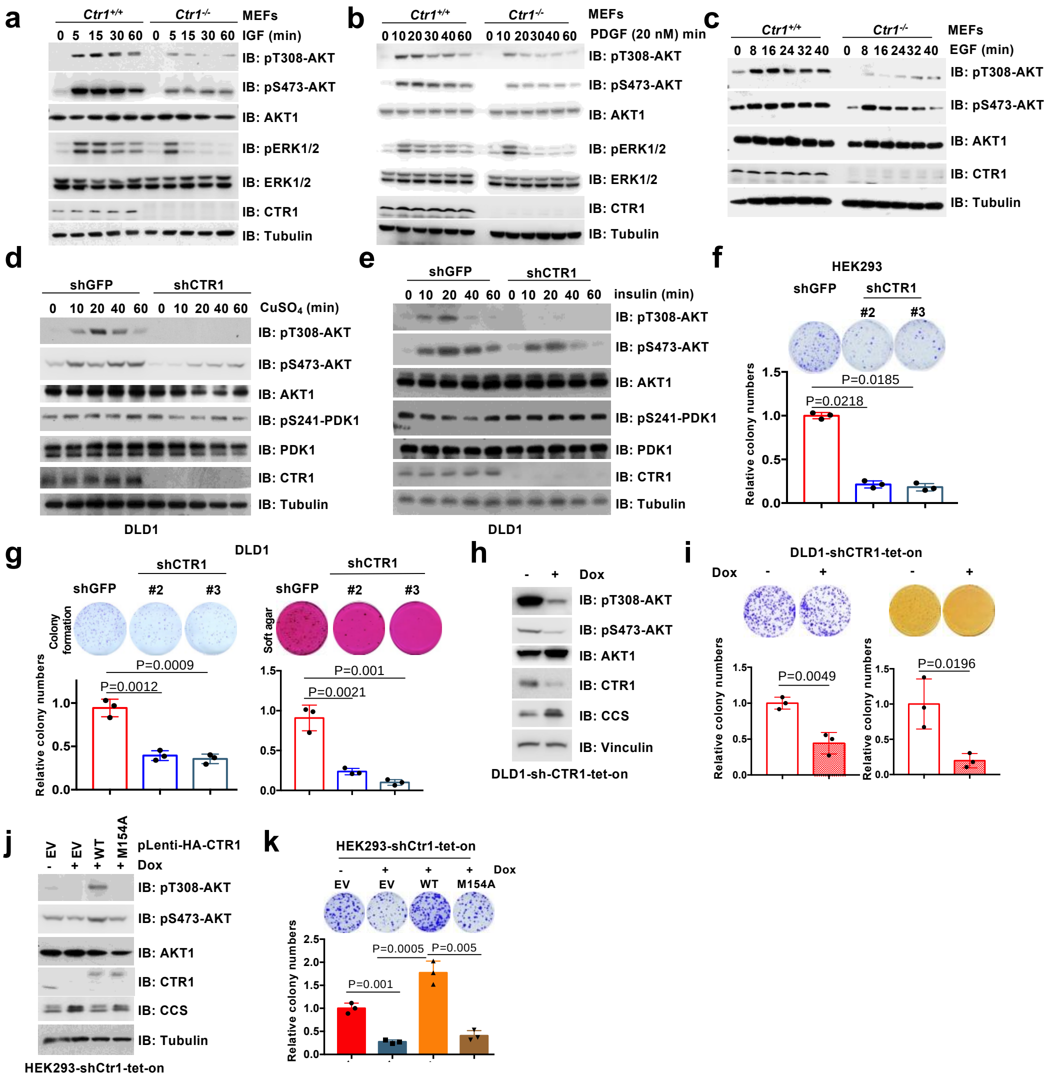

Figure S2. Depleting *CTR1* inhibits cancer cell malignancies. a-c) *Ctr1*-null or WT mouse embryonic fibroblasts (MEFs) were cultured in 1% serum-containing medium for 12 hrs and stimulated with a time course of IGF (0.1 µM) (a), PDGF (20 nM) (b) and EGF (100 ng ml^{-1}) (c) for IB analysis. d,e) DLD1 cells infected with shRNAs against *CTR1* or *GFP* (as a negative control) were stimulated with copper (50 µM) (d) or insulin (0.1 µM) (e) after cultured in 1% serum-containing medium for 12 hrs, and subjected for IB analysis. f) HEK293 cells infected with indicated shRNAs against *CTR1* were subjected for colony formation assays. g) DLD1 cells generated in (d) were subjected to colony formation and soft agar assays (g, top panel). The relative colony numbers were normalized and plotted (g, bottom panel). h,i) DLD1 cells were infected with pLKO.1-Tet-on-sh*CTR1*, and selected with puromycin for 5 days before addition of doxycycline (1 µg ml^{-1}) for 2 days and harvested for IB analysis (h). Resulting cells were subjected to colony formation and soft agar assays (i) (mean ± SD, n = 3) (*t* test). j,k) HEK293 cells were infected with pLKO.1-tet-on-sh*CTR1*, and selected with puromycin for 5 days and infected with viruses encoding indicated proteins selected with hygromycin (100 µg ml^{-1}) for another 3 days before addition of doxycycline for 2 days and harvested for IB analysis (j). Resulting cells were subjected to colony formation assays (k) (mean ± SD, n = 3) (*t* test).

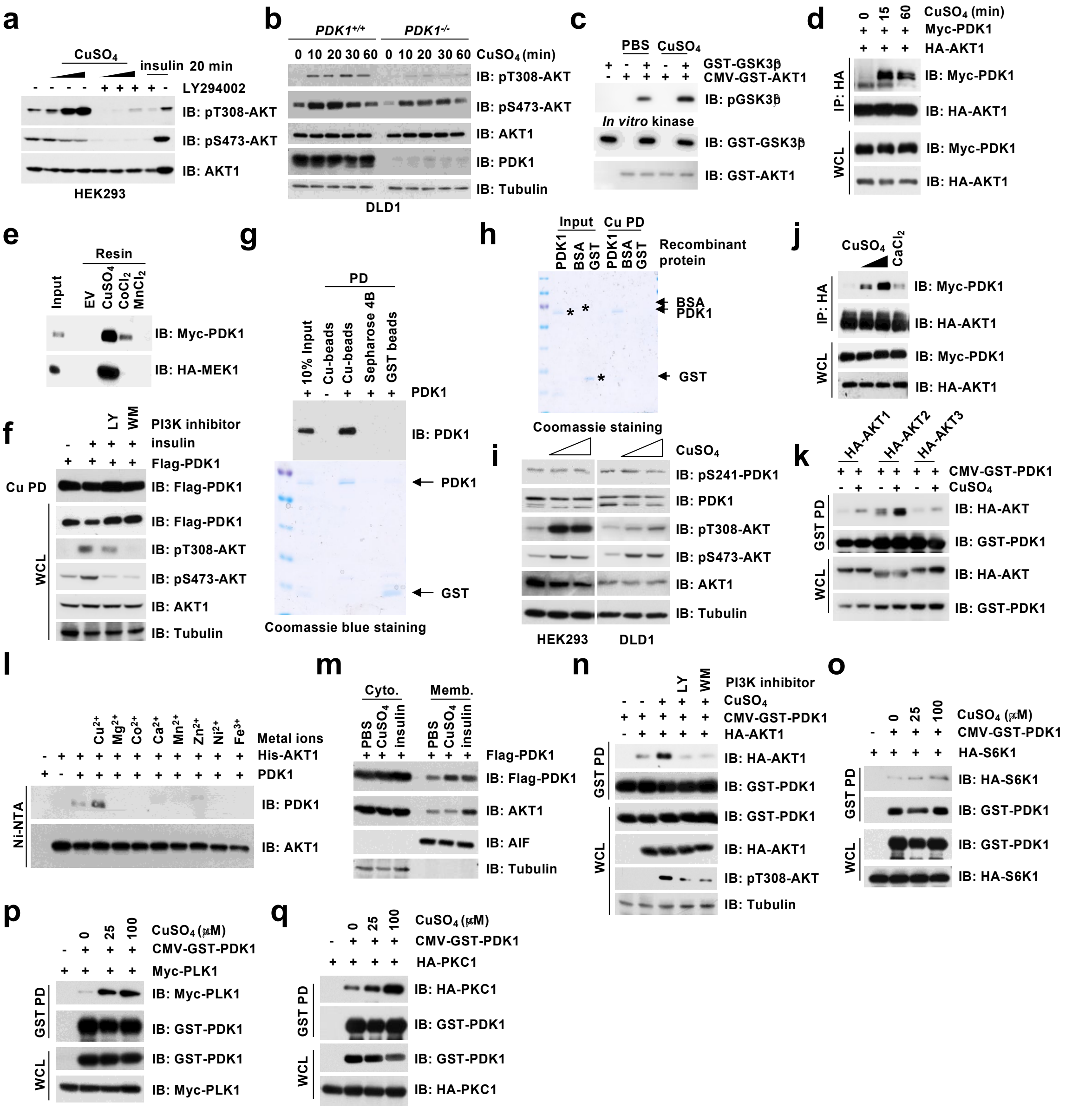

Figure S3. Copper directly binds PDK1 in cells and *in vitro*. a) HEK293 cells were serum-starved for 12 hrs, and treated with LY294002 for 1 hr before stimulated with copper (20 μM) or insulin (0.1 μM) for 20 min. Resulting cells were harvested for IB analysis. b) *PDK1*$^{-/-}$ and parental DLD1 cells were stimulated with a time course of copper (20 μM) after serum-starvation before harvested for IB analysis. c) AKT *in vitro* kinase assays were performed with purified GST-AKT1 from HEK293 cells treated with copper or PBS (as a negative control) after serum starvation as the source of kinase, and the bacterially purified GST-GSK3β was used as the substrate. d) Ni-NTA agaroses conjugated with different metal ions were used to pull down WCL derived from HEK293T cells transfected with indicated proteins and subjected for IB analysis. e) IB analysis of WCL and HA-IP products derived from HEK293 cells treated with a time course of copper after serum starvation 12 hrs. f) Copper pulldown assays were performed with WCL derived from HEK293 cells transfected with indicated constructs and stimulated with Insulin before treated with indicated inhibitors. The pulldown products and WCL were subjected for IB analysis. g) Pulldown assays were performed with beads including

copper-PEG, Sepharose 4B and glutathione Sepharose 4B and the recombinant PDK1 purified from insect cells. The pulldown products were subjected for IB analysis or Coomassie staining. h) Copper pulldown assays were performed with the recombinant proteins including PDK1, BSA and GST. The pulldown products were subjected for Coomassie staining. i) HEK293 and DLD1 cells were treated with different doses of copper after overnight serum starvation before subjected for IB analysis. j) IB analysis of WCL and HA-IP products derived from HEK293 cells treated with copper or calcium after serum starvation 12 hrs. k) HEK293 cells transfected with indicated constructs and stimulated with or without copper (30 μM) after overnight serum-starvation before harvested for GST-pulldown and IB analysis. l) Insect cell purified PDK1 and his-AKT1 proteins were subjected for Ni-NTA pulldown analysis in the presence of various metal ions. The pulldown products and WCL were subjected for IB analysis. m) HEK293 cells transfected with indicated constructs were stimulated with copper (20 μM) or insulin (0.1 μM) for 30 minutes before serum-starvation overnight. Resulting cells were subjected to cell fractionation and IB analysis. n-q) GST pulldown assays and IB analysis were performed with WCL derived from HEK293 cells transfected with indicated constructs and stimulated with copper before treated with or without the indicated inhibitors.

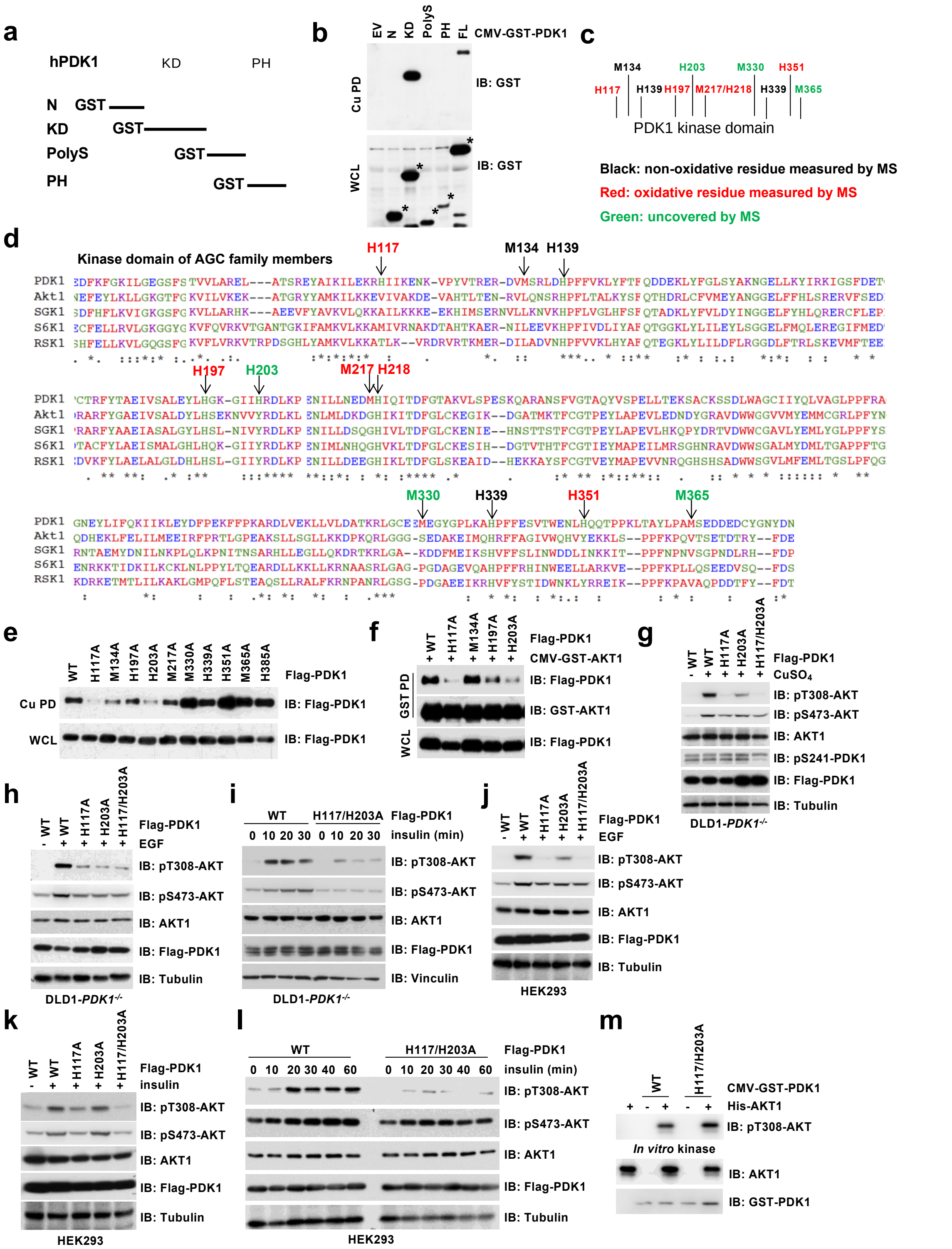

Figure S4. The H117 and H203 residues are identified to be likely the major copper binding residues in PDK1. a) A schematic graph represents the GST fusion fragments of PDK1. b) IB analysis of copper-pulldown products and WCL derived from HEK293T cells transfected with indicated constructs. c) A schematic graph represents the mass spectrometry results derived from *in vitro* oxidative assays in the presence of copper. As indicated, histidine and methionine residues in PDK1 kinase domain were characterized as non-oxidative residue (black), oxidative residue (red) and uncovered (green) in the assays. d) Alignment of the kinase domain of AGC family proteins including PDK1, AKT1, SGK1, S6K1 and RSK1 represents the potential

copper binding residues. e,f) IB analysis of copper-pulldown (e) or GST-pulldown (f) products derived from HEK293T cells transfected with indicated constructs. g-l) DLD1-*PDK1*$^{-/-}$ (g-i) or HEK293 (j-k) cells infected with lentivirus encoding PDK1 mutants were selected with puromycin (1 μg ml^{-1}) for 5 days and stimulated with copper (30 μM), EGF (100 ng ml^{-1}), insulin (0.1 μM) or IGF (0.1 μM) 30 minutes as indicated after overnight serum starvation. Resulting cells were harvested for IB analysis. m) PDK1 *in vitro* kinase assays were performed with the purified GST-PDK1 from HEK293 cells transfected with the indicated constructs as the source of kinase. The insect cell purified His-AKT1 served as substrate. The reaction products were subjected to IB analysis with indicated antibodies.

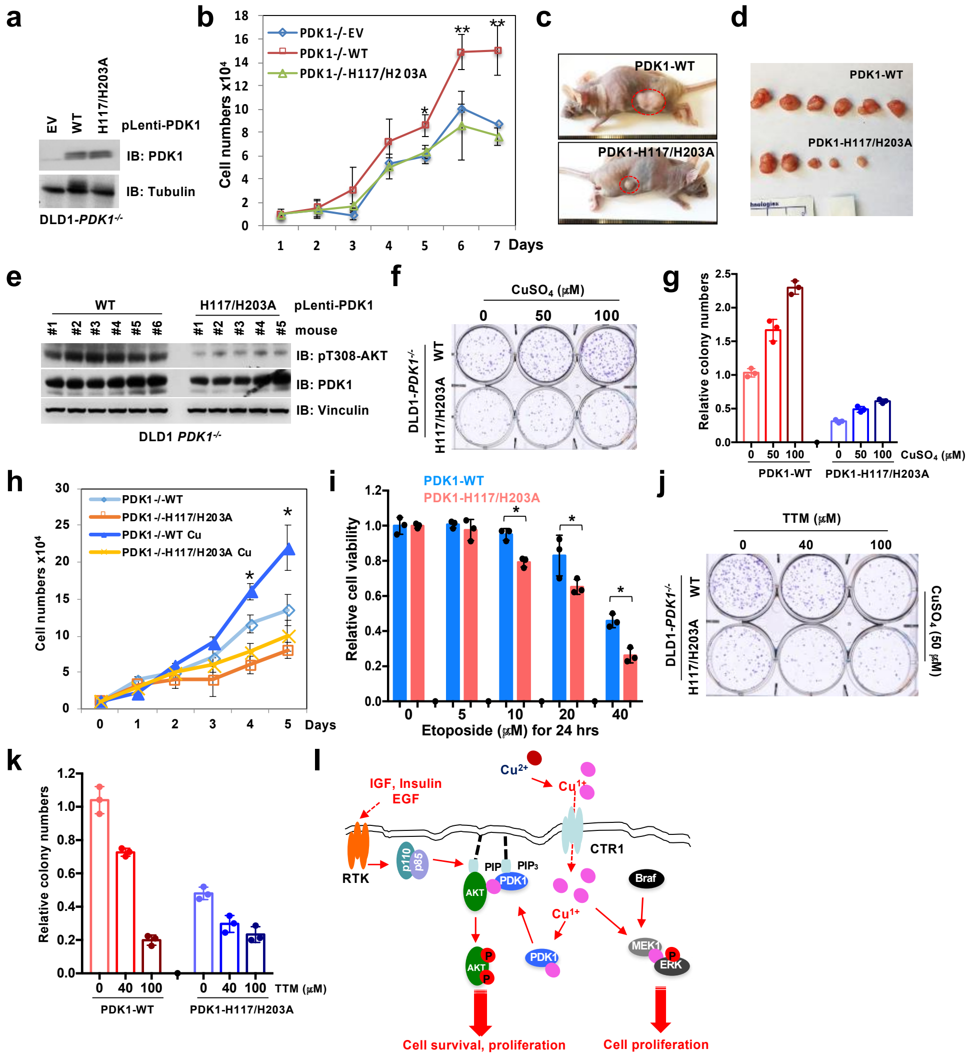

Figure S5. Copper binding deficient mutant form of PDK1 displays decreased ability to activate AKT oncogenic functions. a,b) DLD1-*PDK1*$^{-/-}$ cells infected with lentivirus encoding PDK1 mutants were selected with puromycin (1 μg ml^{-1}) for 5 days and subjected for IB analysis (a), and then the cell numbers were calculated (mean ± SD, n = 3) (*t* test). *P < 0.05, **P < 0.01. (b). c-e) Cells generated in (a) were used for xenograft assays (2 x 10^{6} cells were injected), and the mice were sacrificed once the tumors reached around 1.5 cm (c). The dissected tumors were represented (d), and the lysates derived from these tumors were subjected for IB analysis (e). f-h) Cells generated in (a) were treated with different doses of copper in the medium containing 1% serum, and subjected for colony formation (f) and cell number calculation (h) assays (mean ± SD, n = 3) (*t* test). *P < 0.05. Relative colony numbers were normalized in (g). i) Cells generated in (a) were treated with different doses of Etoposide and subjected to cell viability assays. The relative cell viability was normalized and plotted. (mean ± SD, n = 3) (*t* test). *P < 0.05. j,k) Cells generated in (a) were cultured in the medium containing 1% serum and copper (50 μM), and treated with TTM for 24 hrs and then subjected for colony formation assays (j). Relative colony numbers were normalized (k) (mean ± SD, n = 3). l) A schematic illustration of the proposed model demonstrating that via CTR1, copper ions are transported into cells and bind with oncoproteins such as PDK1, leading to enhanced PDK1 binding and activating its substrates including AKT to promote cellular malignancies.

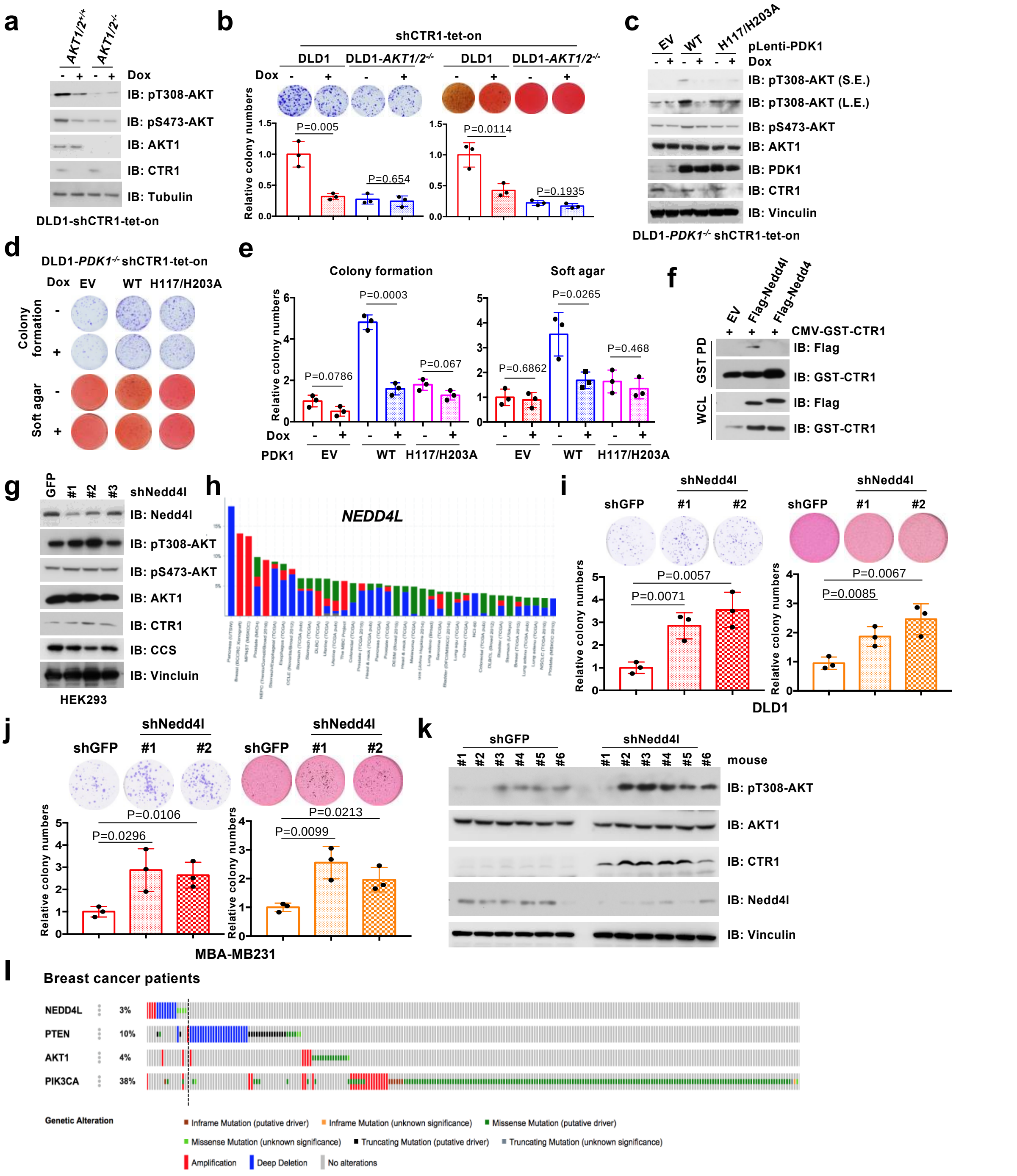

Figure S6. Nedd4l functions as a potential tumor suppressor. a,b) *AKT1/2*$^{-/-}$ and parental DLD1 cells were infected with tet-on inducible shRNAs against *CTR1* and selected with puromycin (1 μg ml^{-1}) for 72 hrs, and then treated with or without doxycycline (1 μg ml^{-1}) for 24 hrs for IB analysis (a). Resulting cells were subjected for colony formation and soft agar assays (b, up panel). The relative colony numbers were normalized and plotted (b, bottom panel) (mean ± SD, n = 3) (*t* test). c-e) *PDK1*$^{-/-}$ DLD1 cells were infected with WT and H117/H203A-PDK1, and selected with hygromycin (200 μg ml^{-1}) for 5 days. Resulting cells were infected with tet-on inducible shRNAs against *CTR1*, and selected with puromycin (1 μg ml^{-1}) for another 5 days and treated with or without doxycycline (1 μg ml^{-1}) for 24 hrs for IB analysis (c), colony

formation and soft agar assays (d). The relative colony numbers were normalized and plotted (e) (mean ± SD, n = 3) (*t* test). f) IB analysis of GST pulldown products and WCL derived from HEK293T cells transfected with indicated constructs. g) HEK293 cells infected with shRNAs against *NEDD4L* were selected with puromycin for 5 days and subjected for IB analysis. h,l) Bioinformatic analyses of genetical alterations of *NEDD4L* in different tumors (h) and their association with the genomic alterations of PI3K-AKT pathway (including the amplification, mutation or deletion of *PIK3CA*, *AKT1* and *PTEN*) via mining the TCGA database (l). i,j), Cells generated in (Figure 5e) were subjected to colony formation and soft agar assays (i,j, top panels). The relative colony numbers were normalized and plotted (i,j, bottom panel) (mean ± SD, n = 3) (*t* test). k) MDA-MB231 cells generated in (Figure 5e) were planted into the flank of nude mice. The tumors were harvested, and the indicated representative harvested tumors were subjected to IB analysis.

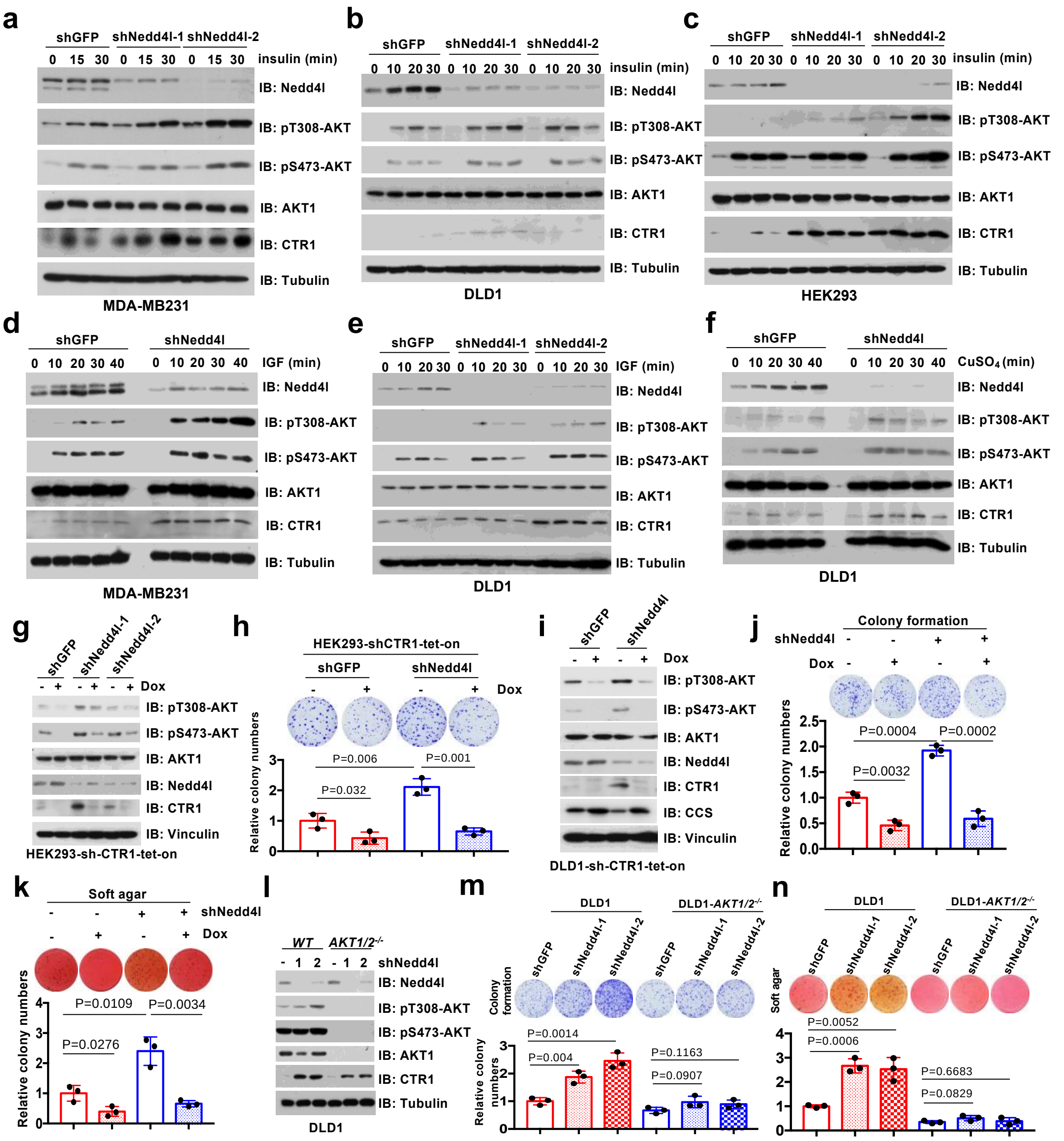

Figure S7. Depleting *NEDD4L* increases AKT kinase activity and CTR1 protein abundance. a-f) MDA-MB231 (a,d), DLD1 (b,e,f) and HEK293 (c) cells infected with shRNAs against *NEDD4L* were selected with puromycin (1 μg ml^{-1}) for 5 days and stimulated with insulin (0.1 μM), copper (30 μM) or IGF (0.1 μM) respectively, and subjected to IB analysis. g-k) HEK293 (g,h) and DLD (i-k) cells were infected with shRNA against *NEDD4L*, and selected with puromycin (1 μg ml^{-1}) for 5 days. The cells were further infected with tet-on inducible shRNAs against *CTR1*, and selected with puromycin (2 μg ml^{-1}) for another 5 days. Resulting cells were treated with or without doxycycline (1 μg ml^{-1}) for 24 hrs and subjected to colony formation and soft agar assays (h,j,k, top panel), the relative colony numbers were normalized and plotted (h,j,k bottom panel) (mean ± SD, n = 3) (*t* test). l-n) *AKT1/2*$^{-/-}$ and WT DLD1 cells were infected with shRNAs against *NEDD4L* and selected with puromycin (1 μg ml^{-1}) for 72 hrs for IB analysis (l). Resulting cells were subjected to colony formation and soft agar assays (m-n, top panel). The relative colony numbers were normalized and plotted (m-n, bottom panel) (mean ± SD, n = 3) (*t* test).

Full unedited gels

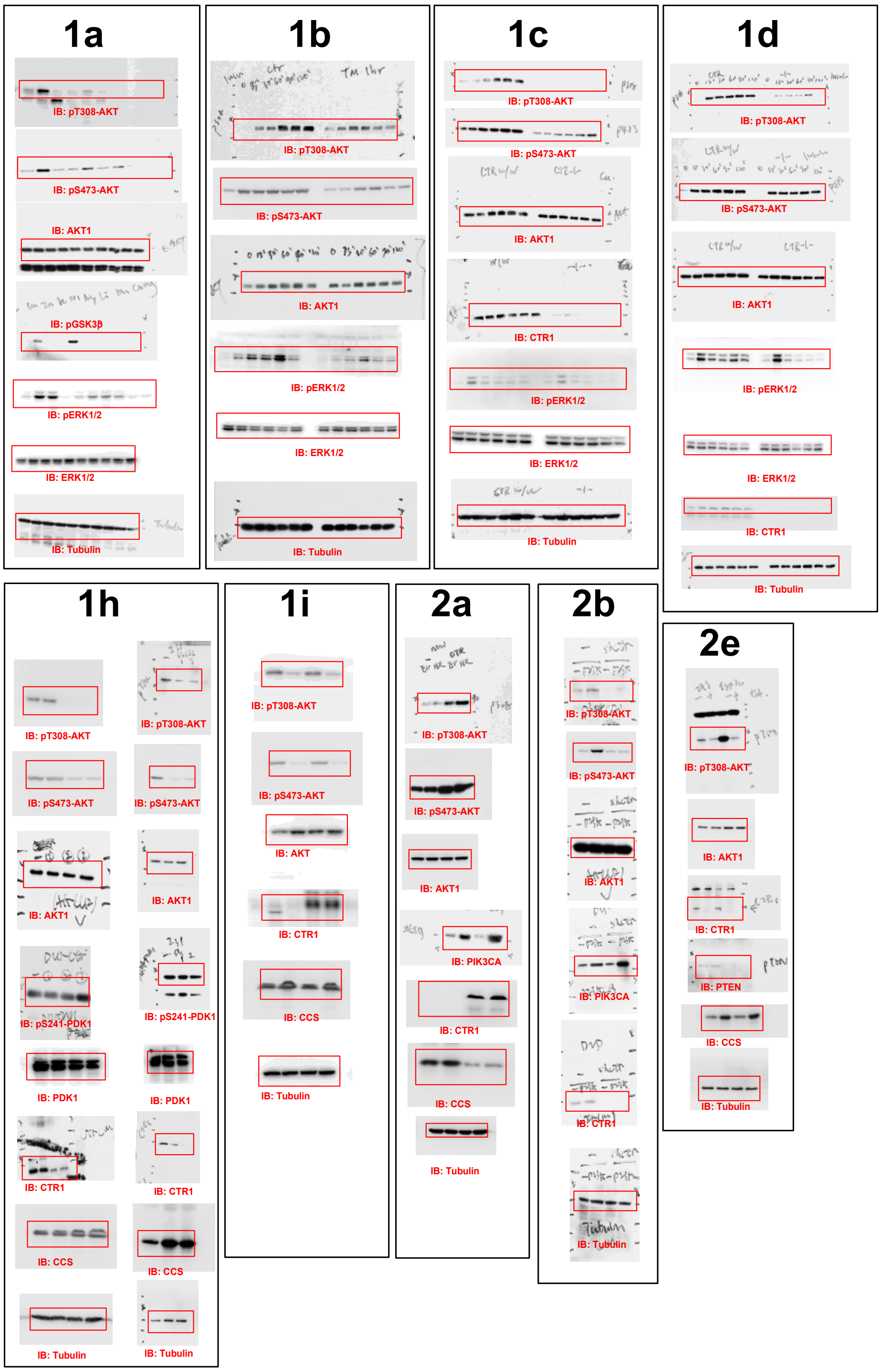

Full unedited gels

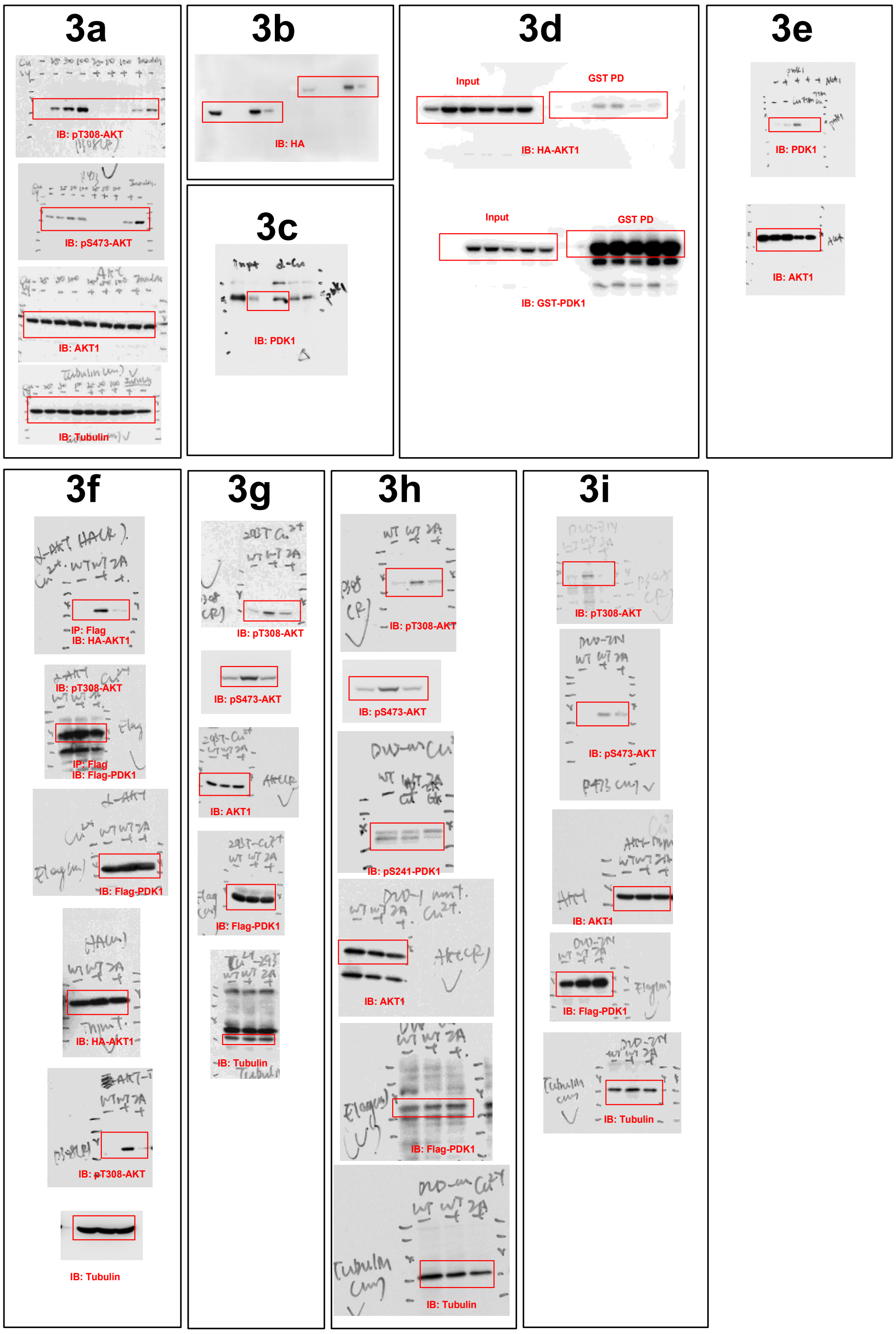

Full unedited gels

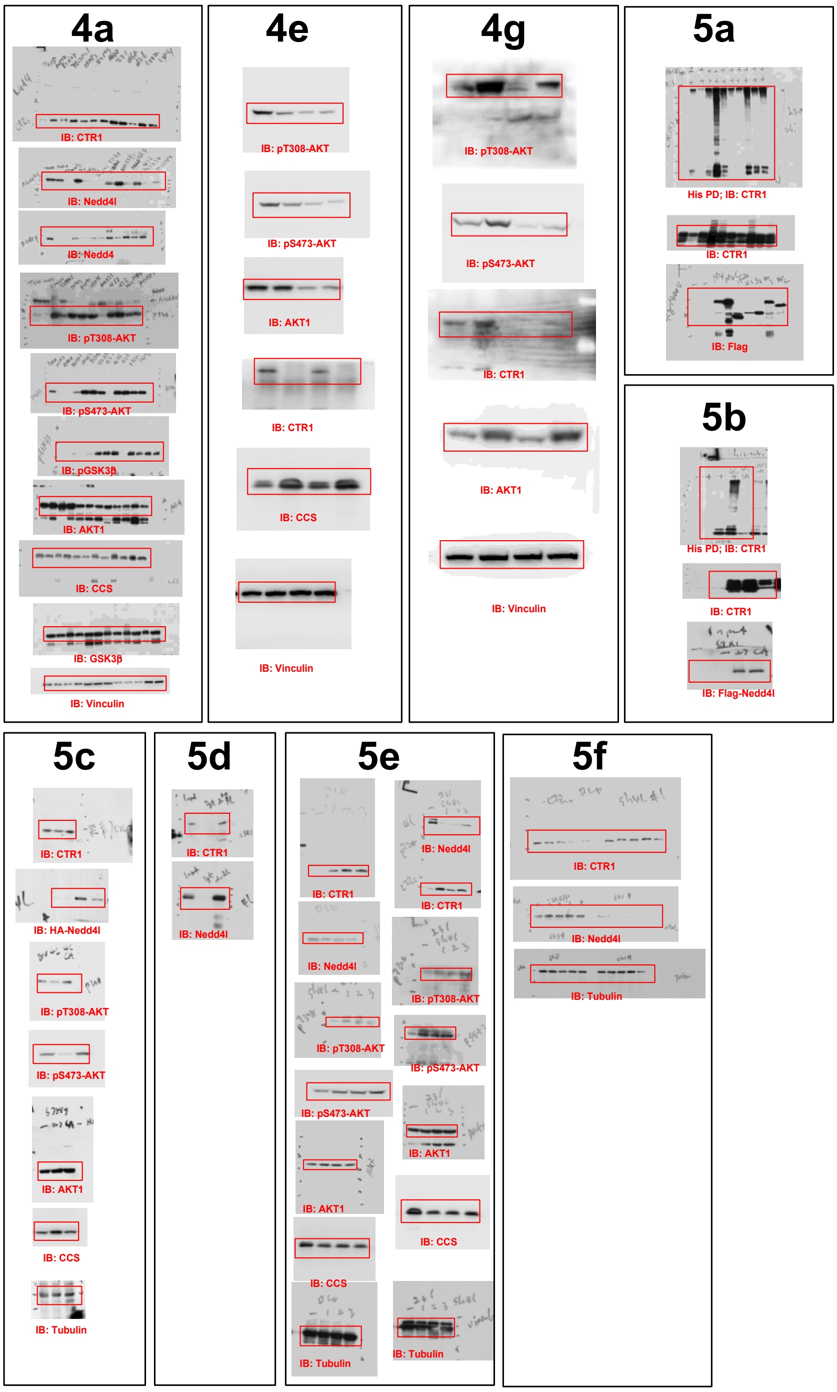

Full unedited gels

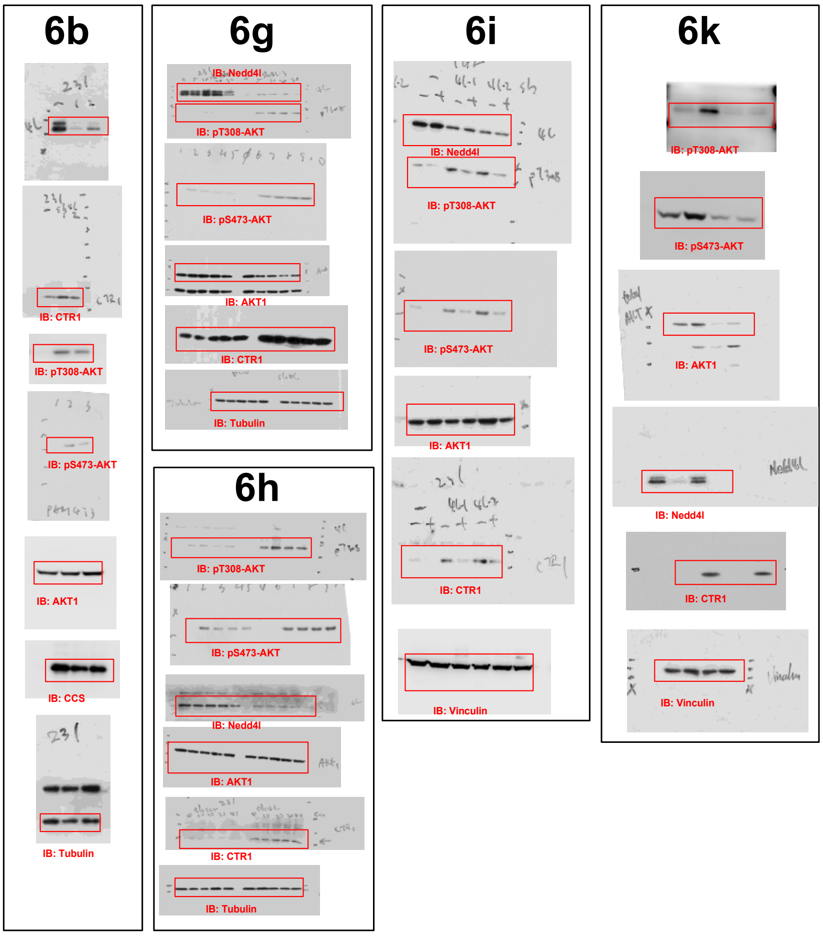

Full unedited gels

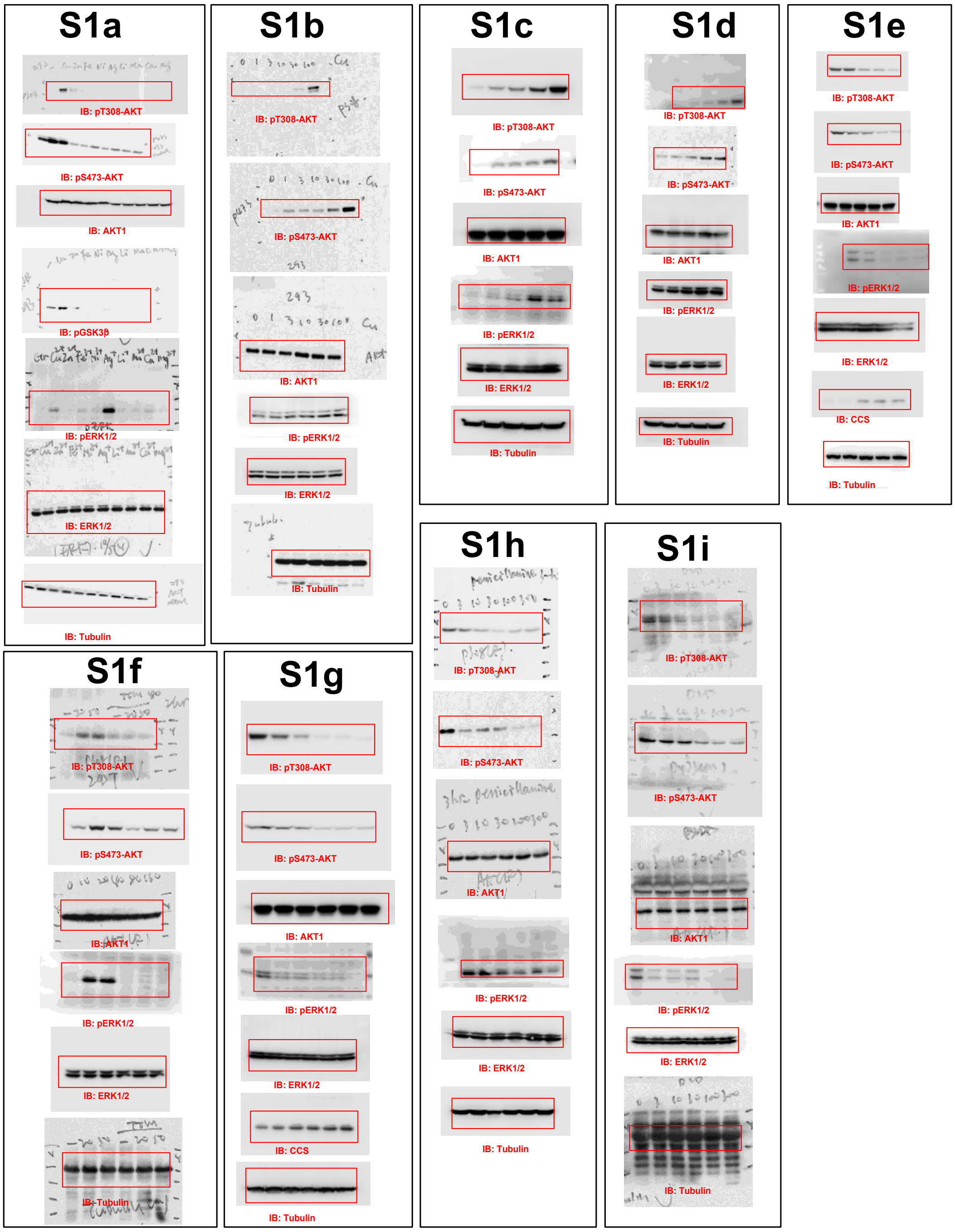

Full unedited gels

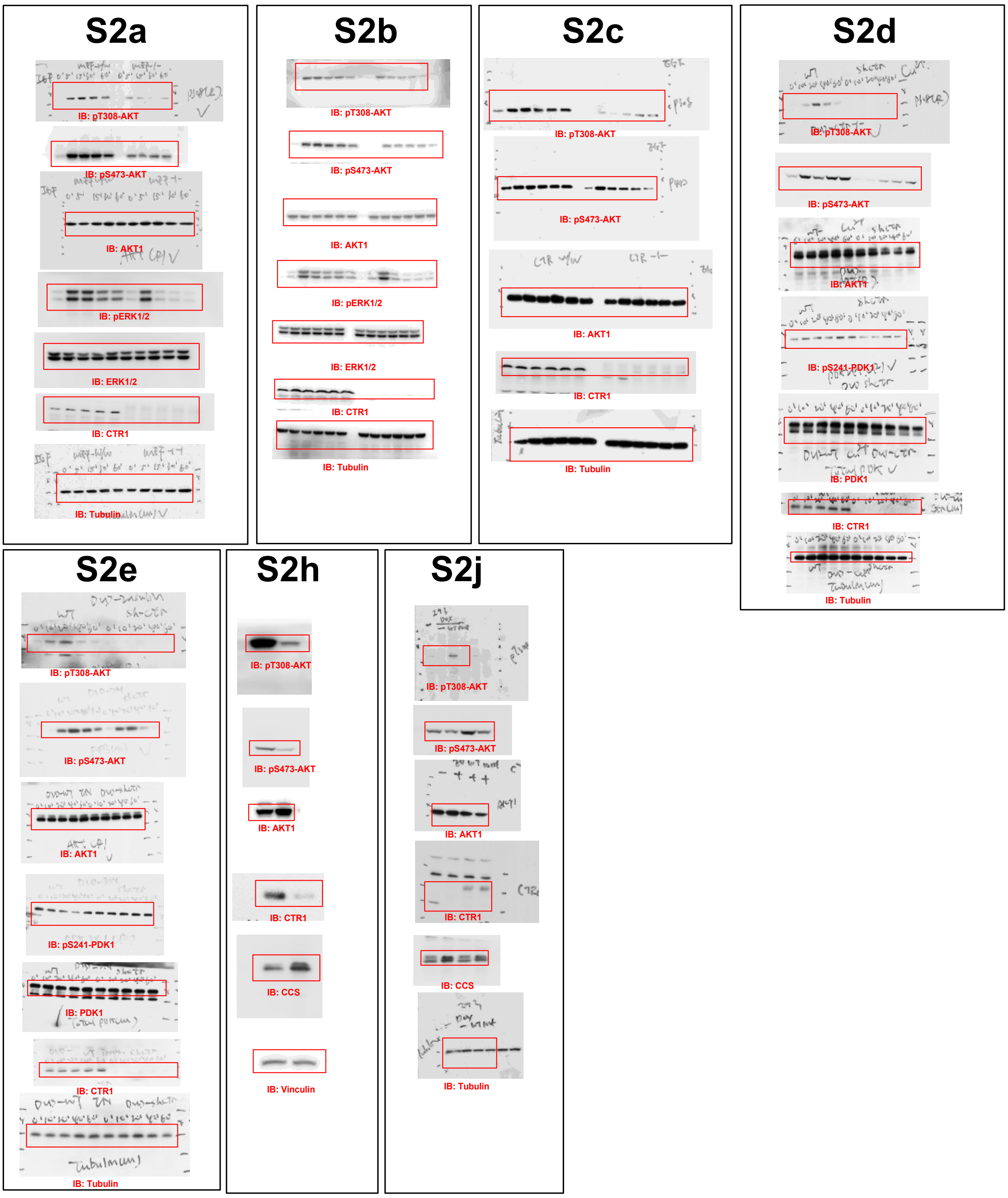

Full unedited gels

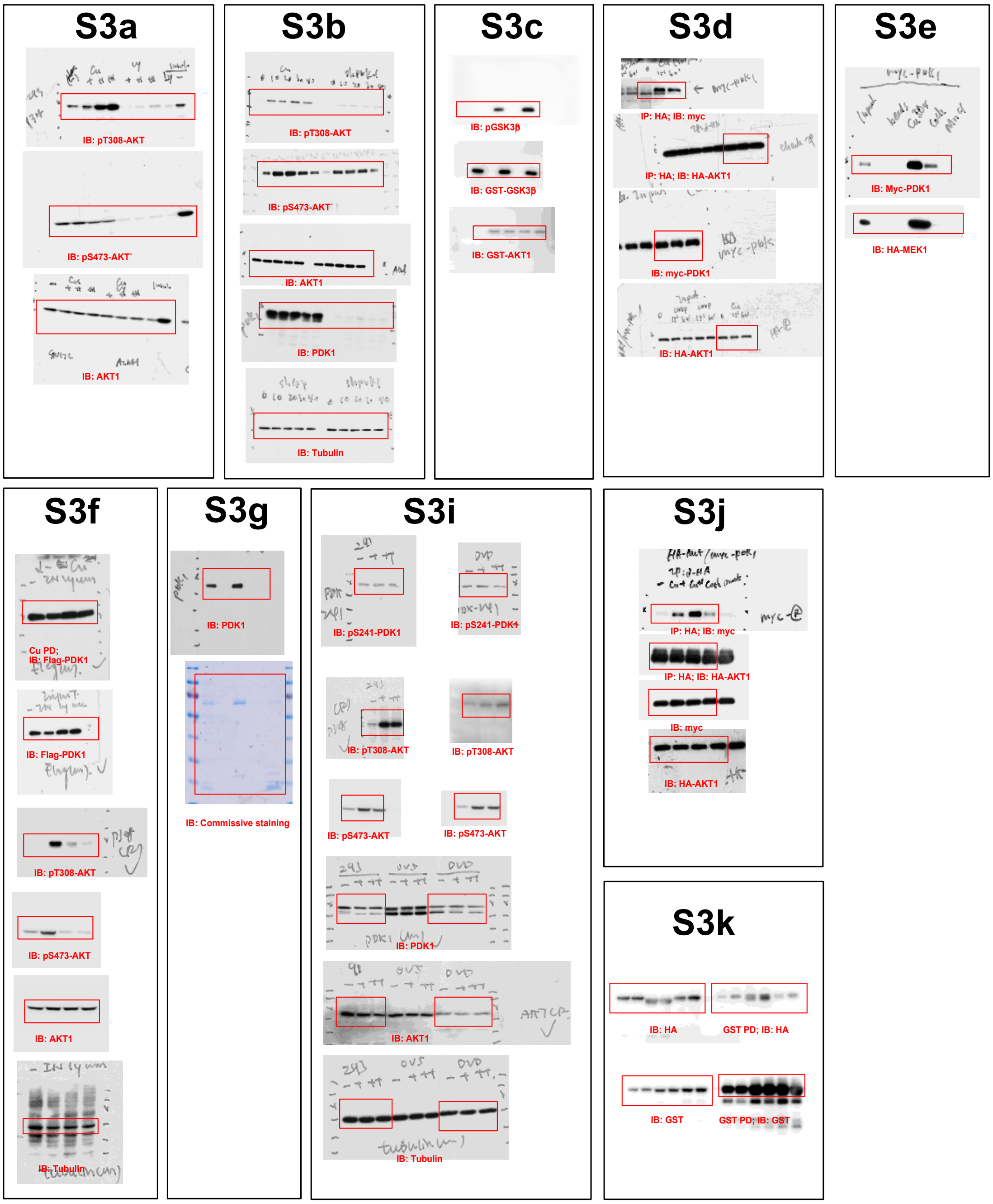

Full unedited gels

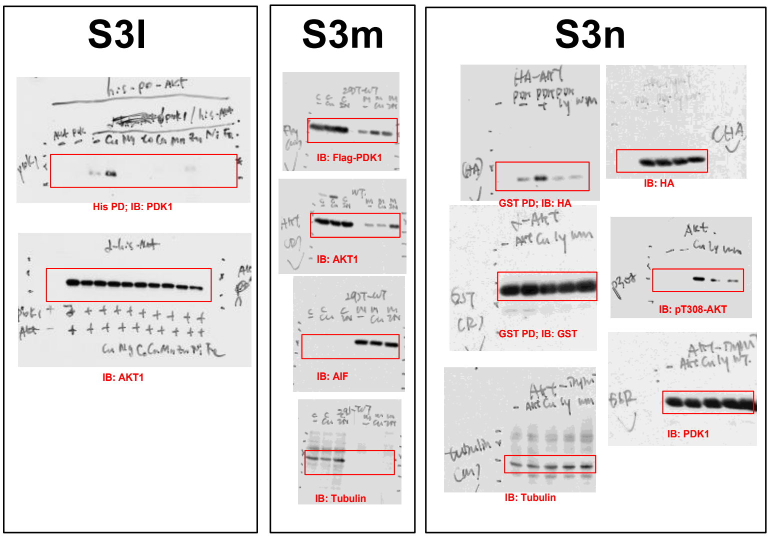

Full unedited gels

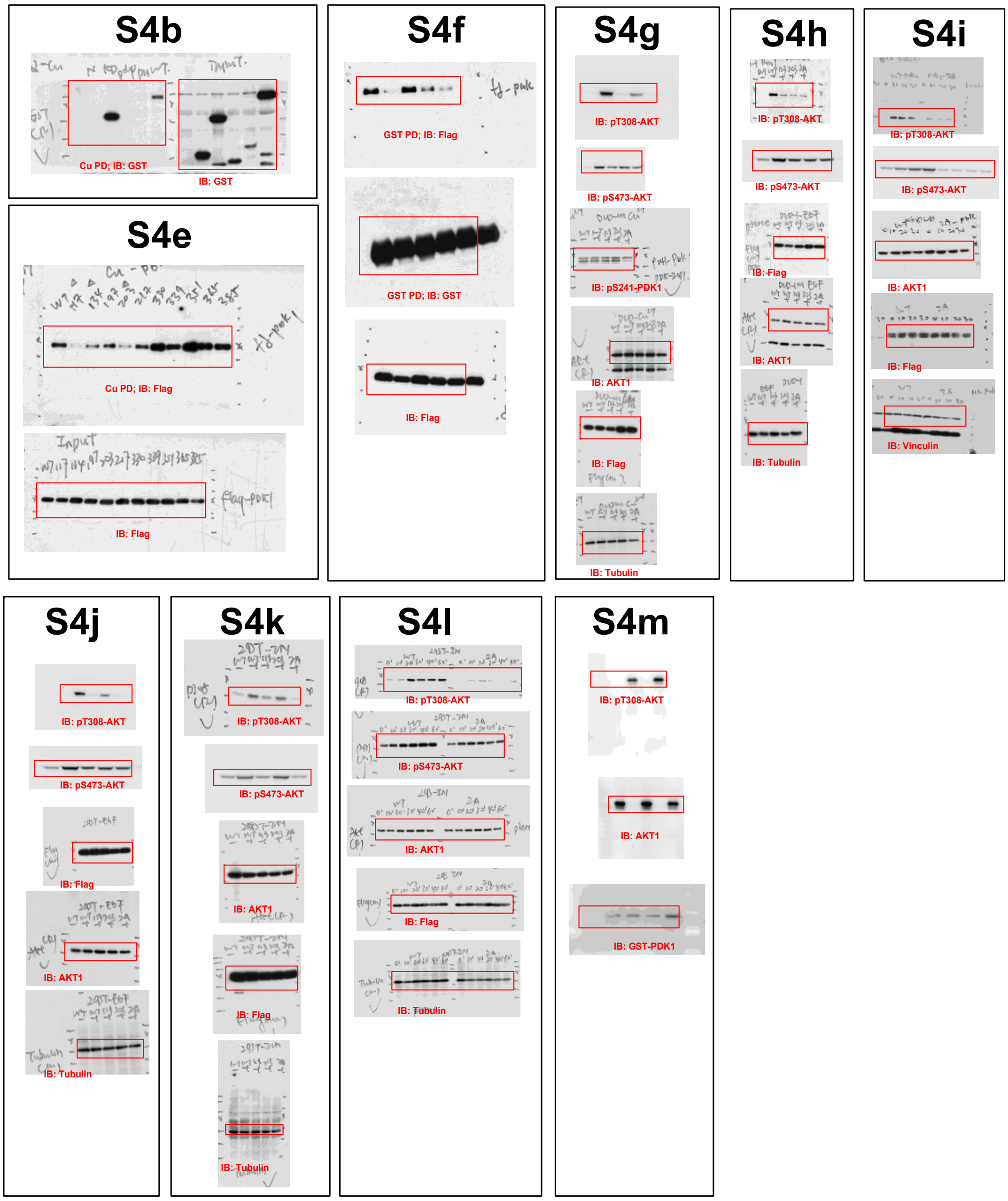

Full unedited gels

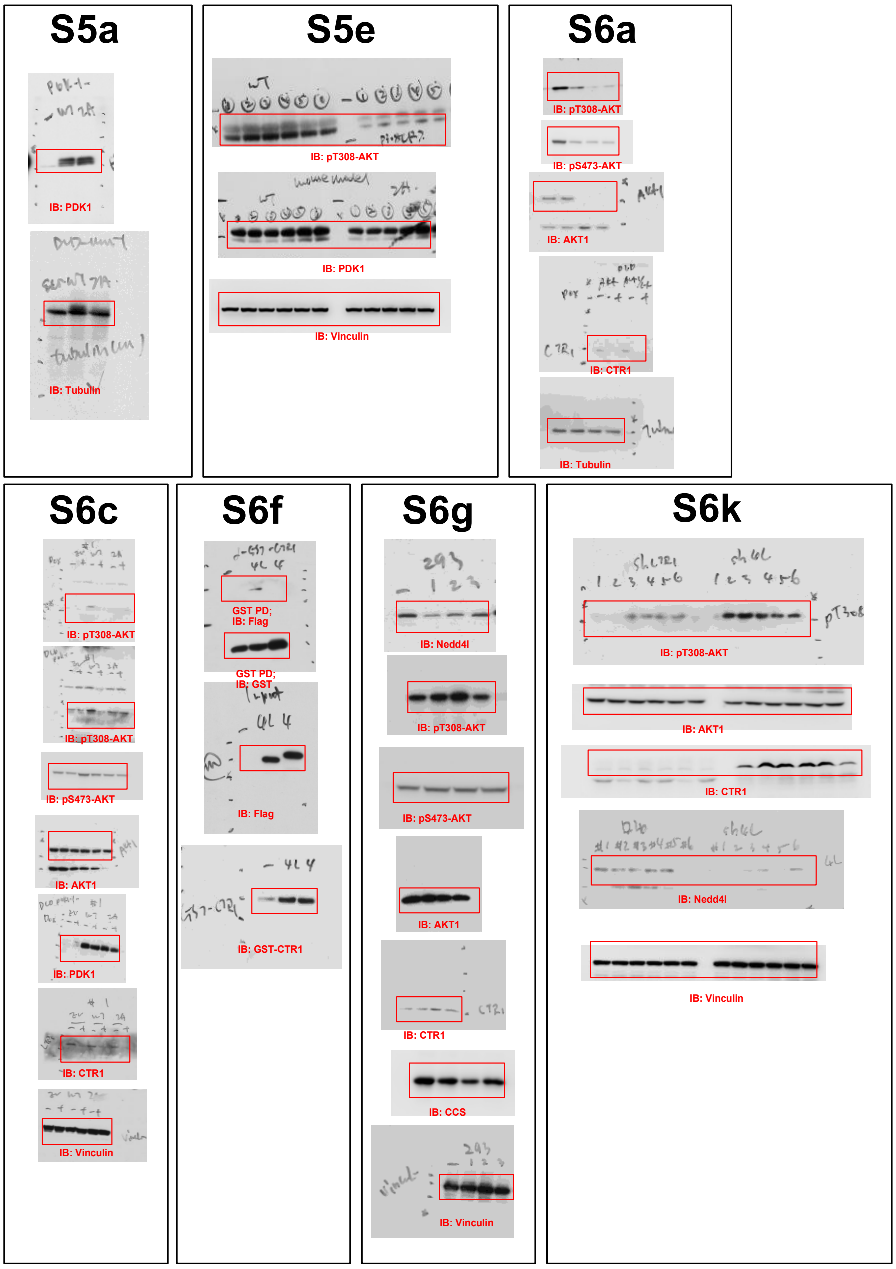

Full unedited gels

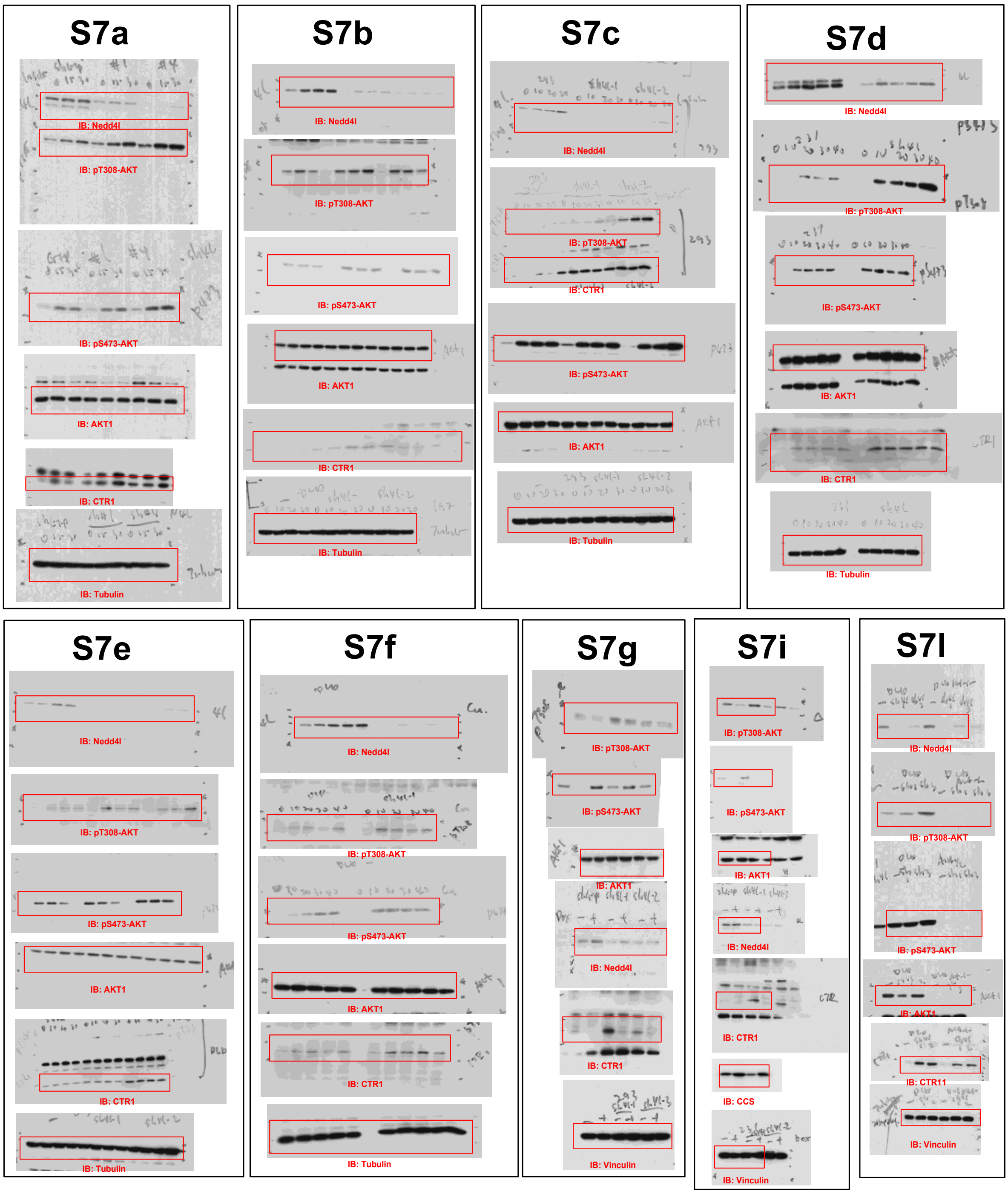

Supplement: Supplementary file 1 — Supporting Information [file ADVS-8-2004303-s002.pdf]
